# Recent Developments to Cope the Antibacterial Resistance via β-Lactamase Inhibition

**DOI:** 10.3390/molecules27123832

**Published:** 2022-06-14

**Authors:** Zafar Iqbal, Jian Sun, Haikang Yang, Jingwen Ji, Lili He, Lijuan Zhai, Jinbo Ji, Pengjuan Zhou, Dong Tang, Yangxiu Mu, Lin Wang, Zhixiang Yang

**Affiliations:** Ningxia Centre of Organic Synthesis and Engineering Technology, Ningxia Academy of Agriculture and Forestry Sciences, No. 590, Huanghe East Road, Jinfeng District, Yinchuan 750002, China; yhk777@yahoo.com (H.Y.); jjw_526@163.com (J.J.); hll_0322@163.com (L.H.); zhailijuan555@163.com (L.Z.); jinboamy@hotmail.com (J.J.); pengjuanzhou@163.com (P.Z.); tangdongabcd@126.com (D.T.); muyangxiu@126.com (Y.M.); elohim_essaim@163.com (L.W.)

**Keywords:** antibacterial resistance, β-lactamase inhibitor, diazabicyclooctane, boronic acids, avibactam, relebactam, vaborbactam

## Abstract

Antibacterial resistance towards the β-lactam (BL) drugs is now ubiquitous, and there is a major global health concern associated with the emergence of new β-lactamases (BLAs) as the primary cause of resistance. In addition to the development of new antibacterial drugs, β-lactamase inhibition is an alternative modality that can be implemented to tackle this resistance channel. This strategy has successfully revitalized the efficacy of a number of otherwise obsolete BLs since the discovery of the first β-lactamase inhibitor (BLI), clavulanic acid. Over the years, β-lactamase inhibition research has grown, leading to the introduction of new synthetic inhibitors, and a few are currently in clinical trials. Of note, the 1, 6-diazabicyclo [3,2,1]octan-7-one (DBO) scaffold gained the attention of researchers around the world, which finally culminated in the approval of two BLIs, avibactam and relebactam, which can successfully inhibit Ambler class A, C, and D β-lactamases. Boronic acids have shown promise in coping with Ambler class B β-lactamases in recent research, in addition to classes A, C, and D with the clinical use of vaborbactam. This review focuses on the further developments in the synthetic strategies using DBO as well as boronic acid derivatives. In addition, various other potential serine- and metallo- β-lactamases inhibitors that have been developed in last few years are discussed briefly as well. Furthermore, binding interactions of the representative inhibitors have been discussed based on the crystal structure data of inhibitor-enzyme complex, published in the literature.

## 1. Introduction

Although recent studies on environmental multispecies bacterial biofilms outweigh the idea of survival of the friendliest [1], individual microorganisms respond to external stimuli in favor of their survival and transfer the acquired adaptations to their offspring. Thus, the survival stress posed by the antibiotics encountered by the individual microorganism manifests in antimicrobial resistance through several mechanisms [2], the production of β-lactamases (BLAs) being one of them. Hence, the evolution of antimicrobial resistance in terms of BLAs can be traced back to millions of years ago [3,4], far before the introduction of modern age antibiotics. This can be attributed to the production of antibiotic agents [5] in soil microbes such as fungi, bacteria, and actinomycetes that act as their self-defense system [6,7], while the microorganisms themselves are not affected by the produced antimicrobial agents. Nevertheless, the term antibiotic resistance was widely used with the reports of antibiotic resistance against sulfa drugs during 1930s, and the discovery of penicillinase, even prior to the clinical use of penicillin [8]. In the following years, the antimicrobial resistance phenomenon resulted in the golden era of antibiotics [3,9] due to widespread development as well as the uncontrolled use of antibiotics in many countries. As a consequence, pathogenic microorganisms, especially Gram-negative bacteria, now possess acquired resistance in addition to their intrinsic or natural capability of fighting antimicrobial agents, which has thus resulted in the emergence of antibiotic-resistant strains [2,10]. Antibiotic degradation and alteration (Table 1) are two of the several mechanisms [2,8,11,12] that are adapted by these bacteria. Of note, the production of BLA is the prime source of BL antibiotic inactivation via the hydrolytic degradation of the lactam ring of the antibiotic, rendering it ineffective against the bacteria. Over the years, an increasing number of BLAs have been proven to be an additive cause of nosocomial as well as community-acquired bacterial infections. Therefore, it is imperative to address all of the aspects of antibiotic resistance [13], such as accumulating the details of resistance mechanisms, the discovery of new targets, target-specific development of new antibiotics, and last but not least, inhibiting the resistance modes. The complete focus on all of the aspects of antibacterial resistance and the strategies to cope resistance mechanisms is a vast area and is open for discussion. However, we are focusing this review on the synthesis of BLIs, with particular emphasis on the derivatives of diazabicyclooctane and boronic acid. A few more classes of compounds have recently been considered for the inhibition of metallo-β-lactamases (MBLs), and we will discuss them briefly.

**Development of modern antibiotics:** The notion of the “Magic Bullet” and the term “Chemotherapy” by Paul Ehrlich at the beginning of 20th century eventually led to him discovering the first chemotherapeutic drug in 1910. The drug arsphenamine (Salvarsan) and its less toxic derivative neoarsphenamine (Neosalvarsan) remained the sole contributors to the chemotherapeutic arena of a bacterial infection, syphilis, for the next twenty years despite exhaustive efforts to discover other magic bullets, which remained unsuccessful [14,15]. Although widely disseminated in the literature, the early structure of Salvarsan proposed by P. Ehrlich was recently reiterated and revised by Nickolson et al. as the mixture of two arsenic compounds [16]. Inspired by the systematic screening approach introduced by Ehrlich and the quick success of the Salvarsan, Heinrich Horlein at the Bayer company led the investigation of potential synthetic compounds to harness chemotherapeutic regime. The compounds synthesized by Bayer chemists, Josef Klarer and Fritz Mietzsch, were tested in vitro and on mice by Gerhard Domagk against a virulent streptococcal strain *Streptococcus hemolyticus*. The extensive screening of multiple compounds spanning around many years of efforts was finally successful with the advent of the azo dyes, which showed antibacterial potential. The antibacterial effectiveness of these azo dyes was later attributed to the presence of a sulfanilamide group in the azo dyes. Several sulfanilamides were then synthesized and were then recognized as sulfa drugs [3,17], the first class of synthetic antibacterial chemotherapeutics. 

The history of BLs began in 1928, with the accidental discovery of penicillin in a fungus *Penicillium notatum*, which was mistaken by Fleming as *Penicillium rubrum* [14,17]. The complete structure of this molecule was revealed by X-ray crystallographic data in 1949 and confirmed by total synthesis in 1957 [8,17]. Limited-scale mass production for the clinical evaluation and commercial use of penicillin was achieved by the united effort of many research organizations, including pharmaceutical companies in the United Kingdom and United States. The efforts were in fact based on the isolation and purification of the drug from mold cultures of *Penicillium baculatum* in order to save the lives of World War II-affected personnel; nonetheless, the process was not viable on commercial scale. Therefore, the joint program of its production was ceased after the end of the war [14]. With the discovery of penicillinase, the interest of pharmaceutical companies and researchers switched to modifying penicillin into an enzyme-resistant drug, which culminated into the introduction of methicillin, followed by phenethecillin, a first semisynthetic derivative of penicillin, which was marketed in the United States and United Kingdom [17].

The word “antibiotics” was coined by Selman Waksman after a systematic study of soil dwelling microbes that are capable of producing antimicrobial agents in the 1930s. According to Waksman, an antibiotic is a compound made by a microbe to destroy other microbes. His work was instrumental in identifying actinomycetes as the producers of the antimicrobial compounds and paved the path towards golden age of antibiotic discoveries from 1940–1960 [7]. Together with the discovery of penicillin, sulphonamides (sulfa drugs), and microbial natural products as potential therapeutic agents against multiple bacterial infections, two parallel lines of antibiotic discovery programs were in force in the forthcoming decades. Antibiotics originating from semisynthetic methods and natural products of microbial origin were fruitful in yielding structurally variable antibiotic classes [4] such as BLs, tetracyclines, aminoglycosides [18], macrolides [19], glycopeptides [20], and quinolones [21], among others [7]. Nevertheless, the pace of drug discovery slowed down after 1962 and onwards, which was perhaps due to the efforts to tailor existing structural scaffolds, resulting in the successful comprehension of the resistance that originates in bacteria from time to time [22]. 

**β-Lactamases:** BLAs are the enzymes that hydrolyze the β-lactam antibiotics by reacting with the carbonyl group of the lactam ring through acylation and deacylation processes. More than 7000 (http://www.bldb.eu/, accessed on 7 April 2020) BLAs have been discovered in a vast variety of Gram-positive and Gram-negative bacteria, so far. The enzymes have been characterized and categorized into various classes, namely Ambler class A–D, based on their structural similarities in their protein sequences [23]. Ambler classes [24,25] A, C, and D (Table 2) are also collectively referred as serine-β-lactamases (SBLs) because of the catalytic nucleophilic activity of the serine residue, whereas class B lactamases rely on zinc ions for their catalytic efficacy, hence also called as metallo-β-lactamases [23]. An alternative classification was described by Bush–Jacoby–Medeiros on the basis of the substrate of a particular set of enzymes. For example, group 1 includes class C cephalosporinase; group 2 comprises class A and D extended-spectrum BLAs, carbapenemases, and broad-spectrum inhibitor-resistant enzymes; and group 3 consists of MBLs [26]. Group 4 contains those unusual penicillinases that are not inhibited by clavulanic acid [27]. In addition, BLAs resistant to extended-spectrum cephalosporins such as TEM, SHV, CTX-M etc., are also referred as extended-spectrum β-lactamases (ESBLs) (Table 3).

BLAs are expressed in both Gram-positive and Gram-negative bacteria; however, they play a vital role as the prime source of resistance in Gram-negative bacteria. The emergence of the first enzyme in 1940 reported in *Bacillus coli* is now regarded as the class C chromosomal cephalosporinase, AmpC [35]. Since the discovery of these enzymes, new non-susceptible antibiotics have been introduced to cope with resistant microbial strains; however, the resistant strains appearing against each β-lactam antibiotic rendered it ineffective over time [36]. It has been speculated that the extensive and unregulated use of these antibiotics is also responsible for the generation of antibiotic-resistant strains. This speculation has been recently assessed, and it has been demonstrated that the sub-MIC concentration of ceftazidime in the *Escherichia coli*-expressing OXA-48 carbapenemase can induce BLAs variants [37]. 

**Figure 1:**
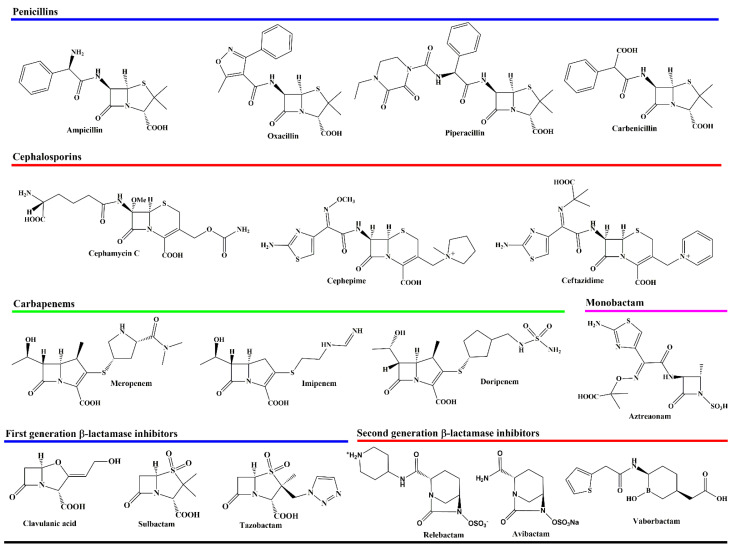
Representative β-lactam antibiotics and β-lactamase inhibitors.

**β-Lactamase Inhibitors:** In addition to the formulation of new resistant antibiotics, an alternative strategy is to inhibit the BLAs so as to safeguard susceptible BL antibiotics. Clavulanic acid is a naturally occurring BL that is isolated from *Streptomyces clavuligerus* [38]. Clavulanic acid is the first-generation BLI that was approved in combination with amoxicillin and ticarcillin [39] for the treatment of urinary tract infections and skin and soft tissue infections. It is available in a solid and liquid forms for oral as well as intravenous administration [40]. Sulbactam is a semisynthetic penicillin-based sulfone BLI with the formula (2S, 5R)-3, 3-dimethyl-7-oxo-4-thia-1-azabicyclo [3.2.0] heptane-2-carboxylate 4,4-dioxide. It is applied in clinics as an intravenous drug in combination with cefoperazone or ampicillin for the treatment of infections caused by BLA-producing *Staphylococcus aureus* and *Moraxella catarrhalis* [38]. In addition to its inhibitory activity, sulbactam also possesses intrinsic antibacterial activity against *Acinetobacter* spp. and *Bacteroides fragilis* [41,42]. Tazobactam is another penicillin-based first-generation BLI that is available together with piperacillin [38] (Figure 1). 

**Diazabicyclooctanes:** The1, 6-diazabicyclo [3,2,1]octan-7-one (DBO) ring was proposed to be the substitute for β-lactam ring by the chemists at the Hoechst Marion Roussel during early 1990s [43]. Although early derivatives of DBO showed poor antibacterial activity compared to traditional BLs, they proved to be weak inhibitors of class A and class C BLAs [43,44]. During the last decade of 20th century, a plethora of diazabicyclic analogs of DBO was prepared by focusing on their BLA inhibition potential and were disclosed in patent applications that began to appear at the beginning of the 21st century [45,46]. These long-term efforts finally proved fruitful with the approval of two non-β-lactam-based BLIs, namely, avibactam and relebactam [44]. The mechanism of action of these second-generation inhibitors is mostly studied using avibactam. It has been realized that avibactam forms a reversible intermediate with the substrate enzyme for its deactivation [47,48]. This is in contrast to its predecessors, clavulanic acid [49], sulbactam, and tazobactam [50], which were suicidal in terms of their mechanism [51] (Figure 1).

**Avibactam:** Avibactam was the first non-BL synthetic BLI approved in combination with ceftazidime in 2015, and later this combination was included with metronidazole in 2018 [39]. These combinations are employed for the treatment of CTX-M, KPC, AmpC, and some OXA enzymes [39,52,53]. The combination of avibactam–ceftazidime has been approved for the treatment of complicated urinary tract infections, complicated intra-abdominal infections, hospital-acquired pneumonia, and other infections caused by aerobic Gram-negative organisms [30]. In addition to these indications, it is also applied for the treatment of hospital-acquired or ventilator-associated bacterial pneumonia in association with metronidazole [39].

The commercial development process of avibactam was started by Novexel and was later taken over by other pharmaceutical companies, and disclosed by Novexel, Aventis, and AstraZeneca/Forest pharmaceuticals. Alternative process development was started by Merck, and the first synthetic route was disclosed in 2009. The optimized development process for avibactam and relebactam has been recently reviewed in detail [48]. The reviews describe the synthetic processes and associated alterations over time in detail and further discuss other avibactam derivatives such as triazole analogues, nacubactam, zidebactam, durlobactam, NXL-105, IID572, ETX2514, and cyclopropane-fused derivatives, etc. [48,54].

**Relebactam:** Relebactam is the second DBO derivative that was approved in combination with imipenem and cilastatin in 2019 [39]. This combination therapy is effective against complicated intra-abdominal infections, complicated urinary tract infections, and hospital-acquired and ventilator-associated bacterial pneumonia, and provides activity against multidrug-resistant *Enterobacterales* isolates expressing ESBLs and KPCs [39].

**Scheme 1:**
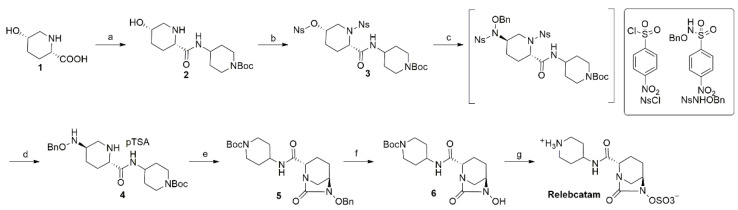
Recent approach for the commercial scale synthesis of relebactam. Reagents and conditions: (**a**) *tert*-butyl 4-aminopiperidine-1-carboxylate, EDC, and HOPO; (**b**) NsCl and DMAP; (**c**) NsNHOBn and K_2_CO_3_; (**d**) HSCH_2_COOH, K_2_CO_3_, and p-TSA; (**e**) CDI and Me_2_SiCl_2_; (**f**) Pd/C, H_2_, BSA, DABCO, and AcOH/H_2_O; (**g**) SO_3_-pyridine, 2-picoline, Bu_4_NHSO_4_, TMSI, and Bu_4_NOAc.

The industrial-scale cost-effective synthesis of relebactam has been developed over the years by Merck [55,56,57,58,59] and has been modified into the recent manufacturing route illustrated in Scheme 1. According to the scheme, commercially available cis-5-hydroxypipecolic acid **(1)** is coupled with Boc-protected piperidineamine to afford the amidation product, which is then protected with nosyl chloride. The resultant product **3** undergoes S_N_2 displacement in bis-4-nosyl amide with benzyloxysulfonamide to afford the intermediate, which is deprotected to form amine **4** as *p*-toluenesulfonic acid salt. The cyclization of amine **4** in the presence of Me_2_SiCl_2_/1,1′-carbonyldiimidazole (CDI) yields benzyl urea **5**, which undergoes palladium (Pd/C)-catalyzed debenzylation in the presence of bis(trimethylsilyl)acetamide (BSA) and 1,4-Diazabicyclo [2.2.2] octane (DABCO) to furnish the hydroxy compound **6**. The hydroxyl group in **6** is reacted with the SO_3_-pyridine complex to form the corresponding sulfonated product. The deprotection of the Boc group is carried out with iodotrimethylsilane (TMSI) in MeCN to ensure that relebactam is the zwitterionic salt [48].

This process, although run on a pilot-scale multiple times, encountered a few shortcomings: (1) oligomer formation was observed in the hydroxy intermediate and in the crude relebactam product, (2) heavy slurry formation at the sulfation step made batch agitation cumbersome, and (3) there were difficulties using TMSI on a pilot scale. Therefore, the Merck team underwent the reinvestigation of these problems and published the outcomes in recent results [60]. It was established that the oligomer formation results from the hydroxy product **6** and is activated by the presence of a base during the reaction and by water in the crystalized form of hydroxy urea product. The optimum reaction conditions required the use of isopropylacetate (IPA) in addition to AcOH and water to suppress oligomer formation during the reaction process. The thickness of the batch during the sulfation reaction was avoided by replacing the SO_3_—pyridine complex with a SO_3_–Et_3_N complex, and TMSI was replaced with bromotrimethylsilane (TMSBr) for the deprotection of Boc in the final step [60].

**Scheme 2:**
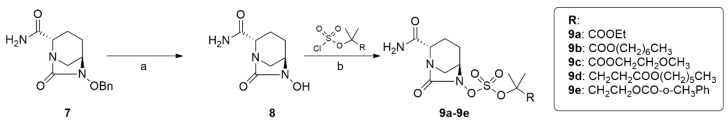
Synthesis of bioavailable avibactam derivatives **9a–9e**. Reagents and conditions: (**a**) Pd/C, H_2_, DMF/DCM, room temperature (rt), and 4 h; (**b**) DBU, DMF, 0 °C-rt, and 16 h.

**Avibactam prodrugs:** In present clinical settings, clavulanic acid [38] is the only BLI that can be administered orally as well as intravenously [61]. In comparison, avibactam, relebactam, and vaborbactam require intravenous inoculation in a hospital for safer administration. Therefore, efforts have been made to ensure the oral availability of BLIs. Avibactam derivatives **9a–9e** for use as avibactam prodrugs, where the sulfate group was protected with variable ester moieties, were prepared (Scheme 2). The compounds were evaluated to determine their bioavailability in animal models and exhibited significant bioavailability in monkey, rat, and dog models [62].

**Figure 2:**
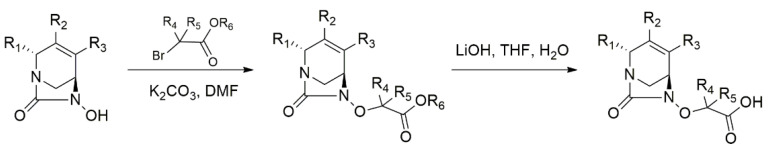
General scheme of synthesis of fluoroacetate-activated durlobactam analogs.

**Durlobactam prodrugs ETX1317 and ETX0282:** Durlobactam is a derivative of avibactam that contains an endocyclic carbon–carbon double bond between C3 and C4, and C4 is substituted with a methyl group [63]. For the bioavailability of this compound, a number of derivatives have been reported, in which the hydroxy sulfation step is replaced with the fluoroacetate group, as an alternative to the sulfonic acid group, at the N6 position [64] of the ring (Figure 2). Of these derivatives, ETX1317, the ester prodrug of ETX0282, has been recognized as a potential orally available durlobactam analog. ETX1317 has exhibited broad spectrum activity against class A, C, and D serine β-lactamases [65] and is currently in clinical trials as an oral therapy for complicated urinary tract infections in combination with cefpodoxime against multiple–drug resistant(MDR) and carbapenem-resistant *Enterobacterales*. Recently, the same research group claimed that the isogenic *Escherichia coli* strains containing the mutated KPC-3 variants that cause resistance to ceftazidime–avibactam do not cause resistance to the combination of cefpodoxime–ETX1317 [66].

**Scheme 3:**
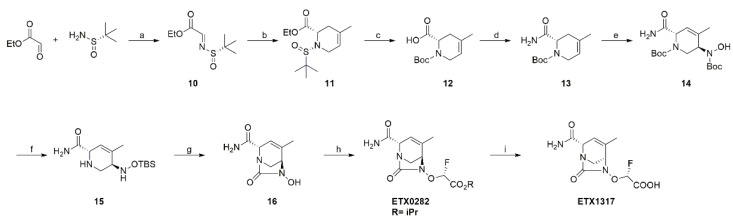
Preparation of durlobactam prodrug ETX0282 and ETX1317. Reagents and conditions: (**a**) DCM, MS 4 Å, 0 °C-rt, and 18 h; (**b**) isoprene, TMSOTf, DCM, −78 °C, and 3 h; (**c**) (i) MeOH, HCl, 0 °C-rt, and 18 h; (ii) NaHCO_3_, Boc_2_O, THF, 0 °C-rt, and 18 h; (iii) LiOH, THF/H_2_O, 0 °C-rt, and 16 h; (**d**) *N,N’*-carbonyldiimidazole, 0 °C, and 5 h then NH_4_OAc, rt, and 18 h; (**e**) BocNHOH, DCM, CuCl, pyridine, O_2_, rt, and 44 h; (**f**) TBSCl, imidazole, DCM, 0 °C, and 18 h then ZnBr_2_, DCM, rt, and 66 h; (**g**) triphosgene, DIPEA, DCM/acetonitrile, 0 °C-rt, and 18 h then HF–pyridine, EtOAc, 0 °C-rt, and 2 h; (**h**) ethyl- or isopropyl- (2*R*)-2-bromo-2-fluoroacetate, DBU, dioxane/DMF, 0 °C, and 10 min; (**i**) LiOH, THF/H_2_O, 0 °C, and 20 min.

The synthesis of ETX0282 was accomplished according to Scheme 3. The key hydroxyurea intermediate **16** was prepared in 10 steps from commercially available ethyl 2-oxoacetate and (*S*)-2-methylpropane-2-sulfinamide. The imine **10** obtained after the condensation of these compounds was cyclized with isoprene through an aza-Diels–Alder reaction to form compound **11**. The deprotection of the *tert*-butylsulfinyl in **11** and the subsequent Boc protection of the intermediate compound furnished **12** in 43% yield after three steps. Ester hydrolysis followed by amidation afforded compound **13** in 62% yield; subsequently, the nitroso ene reaction of alkene **13** with *N*-Boc-hydroxylamine yielded compound **14** regio- and stereoselectively. The chiral compound **14** was then converted to amine **15** by protecting it with the TBS group in first step followed by the removal of Boc with zinc bromide. Compound **15** undergoes cyclization with triphosgene and hydroxy urea derivative **16** is formed after the removal of TBS with HF–pyridine. The hydroxyurea was reacted with ethyl- or isopropyl- (2*R*)-2-bromo-2-fluoroacetate in the presence of DBU to afford the prodrug ETX0282 or corresponding ethyl ester prodrug. ETX1317 (Scheme 3) was prepared in a 63% yield from ETX0282 by saponification with lithium hydroxide [67].

**Scheme 4:**
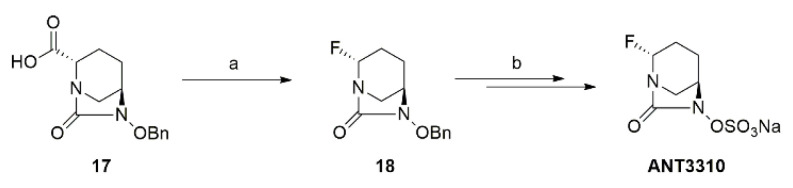
Synthesis of ANT3310. Reagents and conditions: (**a**) AgNO_3_, selectfluor, acetone/H_2_O, 50 °C, and 3 h; (**b**) Pd/C, H_2_, MeOH, rt, and 4 h; SO_3_–pyridine, pyridine, rt, and 3 h; DOWEX^®®^50WX8 Na^+^.

**ANT3310:** ANT3310 is the derivative of avibactam in which the carboxamide group of avibactam has been replaced with fluorine atoms (Scheme 4). This compound has been useful in restoring the carbapenem activity against OXA-CRAB as well as other SBL-carrying carbapenem-resistant *Acinetobacter baumannii* strains in in vivo mouse infection models [68].

**Scheme 5:**
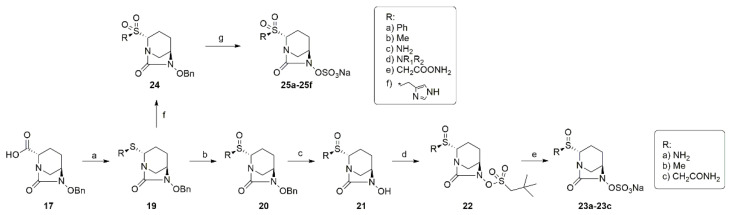
Synthesis of sulfinates and sulfonates from the thioether derivative of DBO. Reagents and conditions: (**a**) 2-mercaptopyridine-1-oxide, EDC·HCl, DCM, thiolating agent, rt, and LED; (**b**) m-CPBA, (1.1 eq), DCM, −78 °C; (**c**) Pd/C, H_2_ (1 atm), and rt; (**d**) neopentyl chlorosulfate, DBU, DMF, and 0 °C; (**e**) sodium 1,3,4-thiadiazole-2-thiolate, MeCN/H_2_O, and rt; (**f**) m-CBPA, (2.2 eq), DCM, rt; (**g**) Pd/C, H_2_ (1 atm), rt, SO_3_-pyridine, pyridine, and rt.

**Thio-substituted DBOs:** Researchers from Shiniogi pharmaceuticals reported a series of thio-substituted DBO derivatives in which the C2 position of DBO was transformed into sulfide, which was then oxidized to sulfinates and sulfones. To access the sulfide derivatives, commercially available carboxylic acid **17** was subjected to a photo-induced Barton decarboxylative thiolation reaction with appropriate thiolating agents in the presence of 2-mercaptopyridine-1-oxide and Ethyl-3-(3-dimethylaminopropyl)carbodiimide hydrochloride (EDC·HCl) [69,70]. The oxidation of sulfide intermediate **19** with m-chloroperoxybenzoic acid (m-CPBA) in different conditions could generate good yields of the respective sulfinates and sulfones. The final products, **23a–23c** and **25a–25f**, were obtained by following the debenzylation and sulfonation process at the N6 position of the DBO ring according to standard procedures (Scheme 5) [69,70].

**Scheme 6:**
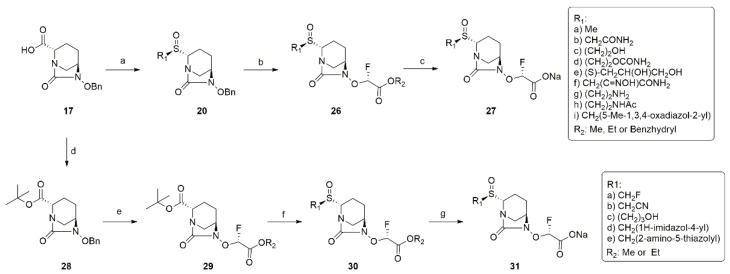
Synthesis of fluoroacetate-activated sulfinates from thioether derivative of DBO. Reagents and conditions: (**a**) 2-mercaptopyridine-1-oxide, thiolating agent, EDC·HCl, DCM, and −50 °C; (**b**) Pd(OH)_2_ on carbon, DABCO, H_2_ (1 atm), and rt; (*R*)-BrCHFCO_2_R_2_, DBU, DMF, 0 °C; (**c**) NaOH, THF/H_2_O, and 0 °C; (**d**) t-BuOH, pyridine, EDC·HCl, DCM, DMAP, and rt; (**e**) Pd(OH)_2_ on carbon, DABCO, H_2_ (1 atm), MeOH or DMF, and rt then (*R*)-BrCHFCO_2_R_2_, K_2_CO_3_, DMF, and −40 to −20 °C; (**f**) TiCl_4_, MeNO_2_, 2-mercaptopyridine-1-oxide, EDC·HCl, DCM, thiolating agent, white LED, and 0 °C; (**g**) NaOH, THF/H_2_O, and 0 °C.

**Scheme 7:**
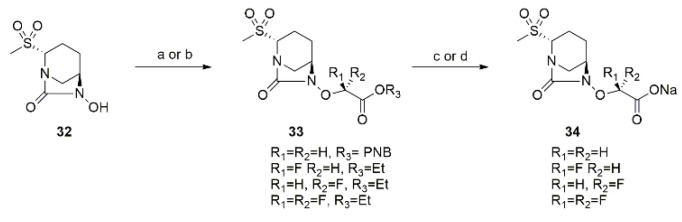
Synthesis of fluoroacetate-activated sulfonates from the thioether derivative of DBO. Reagents and conditions: (**a**) R-X, K_2_CO_3_, DMF, and rt; (**b**) (*R*)-ethyl bromofluoro- or (difluoro)-acetate, DBU, DMF, and 0 °C; (**c**) NaHCO_3_, DMF, and 0 °C; (**d**) NaOH, THF/H_2_O, and 0 °C.

This research group also prepared thio-substituted DBOs in which the *N*-hydroxy position of DBO was activated with fluoroacetate derivatives instead of sulfonic acid. Thus compounds **27** and **31** were synthesized via two different synthetic routes. In one route, thiolation and subsequent oxidation were introduced to the carboxylic acid derivative **17** prior to the deprotection of the benzyl ether. The thioether intermediate was oxidized to **20** followed by debenzylation to form the hydroxy intermediates, which were substituted with the fluoroacetate group. Alternatively, the carboxyl group in **17** was first esterified to form **28**, and appropriate alkyl bromofluoroacetate was reacted to the benzyl deprotected intermediate to form the fluoroacetate derivatives **29**. The individual compounds **29** were then subjected to photo-induced thiolation before saponification in R_2_ to reach the final target compounds **31** (Scheme 6 and Scheme 7) [71].

**Scheme 8:**
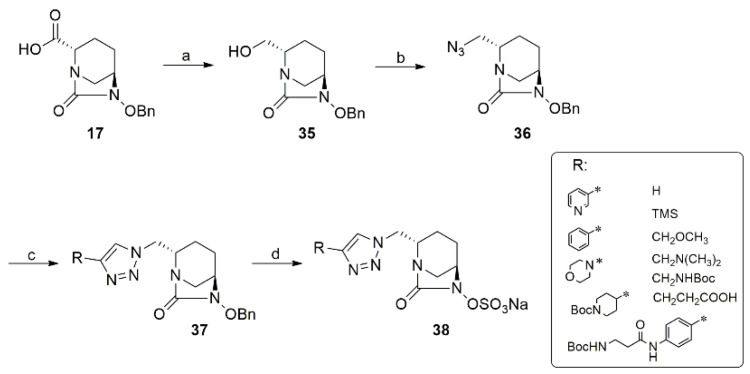
Synthesis of triazole-substituted DBO analogs. Reagents and conditions: (**a**) isobutylchloroformate, TEA, THF, 0 °C, and 2 h then NaBH_4_, 0 °C, and 3 h; (**b**) MsCl, TEA, DMAP, DCM, 0 °C, and 1 h; NaN_3_, DMF, 90 °C, and 20 h; (**c**) alkyne, CuSO_4_ 30 mol %, sodium ascorbate 60 mol %, THF/H_2_O (3/1), rt, 16 h (**d**) Pd/C, H_2_, MeOH, rt, 48 h, SO_3_–pyridine, pyridine, rt, overnight, and DOWEX Na^+^ ion exchange chromatography.

**Triazole functionalized DBOs:** Unsubstituted and several triazole ring-substituted DBO derivatives (Scheme 8) were synthesized for inhibition against TEM-1, KPC-2, CTX-M-15, AmpC, and OXA-48 BLAs in vitro. The unsubstituted triazole derivatives proved to be less effective compared to the substituted ones and the reference compounds, avibactam and relebactam. Of the substituted derivatives, the pyridyl- and phenyl-substituted derivatives showed significant inhibition against KPC-2 carbapenemase and the CTX-M-15 extended-spectrum β-lactamase [72]. The synthesis of the triazole function at the C2 position of the DBO scaffold required the azide derivative **36,** which was initially synthesized in 12 steps starting from the oxopyrrolidine [73]. However, with the commercial availability of the carboxylic acid derivative **17**, modified azide synthesis was achieved in two steps. The compound **17** was first activated with iso-butylchloroformate prior to its reduction to alcohol **35** with NaBH_4_. The alcohol is then activated with mesylchloride, and the thus formed intermediate is substituted with azide to form **36**. The azido derivative **36** undergoes copper(I)-catalyzed Huisgen–Sharpless cycloaddition with the appropriate alkynes to form the corresponding triazoles **37**, which can be transformed into corresponding sulfates **38** by reducing the benzyl ethers and subsequent sulfation followed by ion exchange chromatography [72] (Scheme 8).

**Scheme 9:**
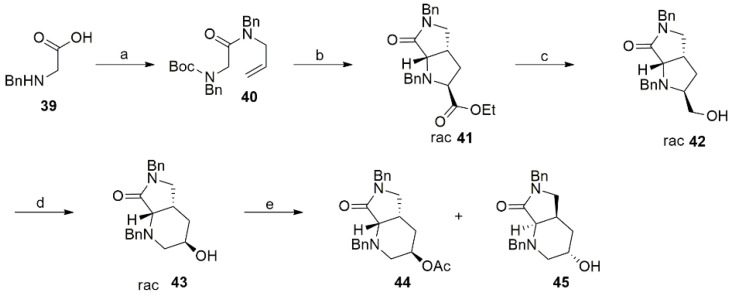
Synthesis of enantiopure intermediate **44**. Reagents and conditions: (**a**) Boc_2_O, THF/H_2_O, rt, and 16 h then allylbenzylamine, HATU, DIPEA, DMF, rt, and 16 h; (**b**) TFA, DCM, rt, 16 h, ethyl 2-oxoacetate, toluene, and 15 h; Et_3_N, MgSO_4_, reflux, and 6 h; (**c**) LiBH_4_, THF, rt, and 6 days; (**d**) TFAA, Et_3_N, and reflux 135 h; NaOH, rt, and 15 min; (**e**) lipase QLM, vinyl acetate, TBME, 30 °C, 6 days, and chromatography.

**Scheme 10:**
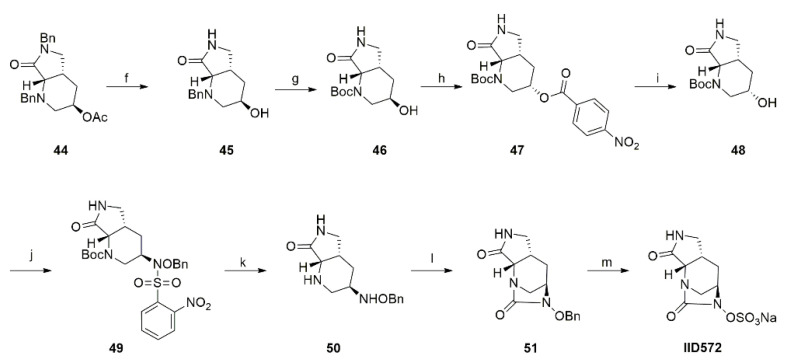
Synthesis of target compound **IID572** from **44**. Reagents and conditions: (**f**) NaOH, THF/MeOH, 25–40 °C, and 65 h; NH_3_(**l**), Li, EtOH, THF, and −78 to −63 °C; (**g**) H_2_, Pd/C, Boc_2_O, MeOH, 22-25 °C, and 89 h; (**h**) 4-nitrobenzoic acid, PPh_3_, DIAD, THF, and −10 to 20 °C; (**i**) K_2_CO_3_, MeOH, 20 °C, and 1 h; (**j**) O-Bn-2-nitrobenzenesulfonamide, DIAD, PPh_3_, THF, 10 °C, and 94 h; (**k**) TFA, DCM, rt, 75 min, PhSH, K_2_CO_3_, MeOH, rt, and 22 h; (**l**) COCl_2_, Et_3_N, CAN, 5 °C, and 4 h; (**m**) H_2_, Pd/C, DCM, MeOH, rt, and 90 min; SO_3_–pyridine, rt, and 18 h then Bu_4_NHSO_4_, aq. NaH_2_PO_4_, DCM, rt, and 30 min; sodium 2-ethylhexanoate, isobutanol/H_2_O, 40 °C, and 1 h.

**Improved Process of IID572:** The carboxamide group in avibactam was replaced with a pyrrolidone ring by Novartis to form a tricyclic DBO called **IID572**. This DBO analog is under development by Novartis in combination with piperacillin. Recent studies have shown that **IID572** effectively protects piperacillin against degradation by serine BLA-expressing drug-resistant *Enterobacteriaceae* and that the combination is effective against many tazobactam/piperacillin-resistant strains [74]. The discovery route for **IID572** started from the carboxylic acid **17** derivative over eight steps to reach the final product with 0.9% overall yield [48,74]. A scale-up synthesis of the compound was recently developed from readily available materials over 19 steps, resulting in an overall yield of 1.7% [75] (Scheme 9 and Scheme 10). According to Scheme 9, the Boc protection of 2-(benzylamino)acetic acid (**39**) was achieved in quantitative yields followed by the coupling of the resultant compound with commercially available allylbenzylamine to obtain compound **40**. The deprotection of the Boc group in **40** and the subsequent coupling with ethyl glyoxylate significantly yields the cycloaddition product **41** as a racemic mixture at a 76% yield. The reduction of the ethyl ester in **41** with LiBH_4_ led to the formation of alcohol **42**. The reaction of **42** with trifluoroacetic anhydride formed the trifluoroacetate analog in situ, which was replaced by benzylamine to form an aziridinium intermediate that was re-opened to access the six-membered ring compound **43** after saponification with aq. NaOH. The separation of the required enantiomer **44** was achieved by QLM lipase in high enantiomeric purity [75].

After the successful isolation of the pure enantiomer **44**, Scheme 10 is followed to convert it to the target compound. Compound **46** is obtained in three steps via the ester saponification of **44** with NaOH followed by debenzylation in two consecutive reactions. Debenzylation at the lactam nitrogen was accomplished under Birch conditions using Li and EtOH to form the intermediate compound **45**. In the next step, the second benzyl group is hydrogenated under standard conditions followed by NH protection with Boc anhydride in the same pot to form derivative **46**. The inversion of the stereo center in **46** was achieved by the formation of the 4-nitrobenzoic ester **47** under Mitsunobu conditions followed by saponification with K_2_CO_3_. Thus, an intermediate alcohol **48** was obtained and then reacted with *N*-(benzyloxy)-2-nitrobenzenesulfonamide to form compound **49,** which, after Boc deprotection, was converted to **51** after cyclic urea formation by phosgene. The benzyl deprotection in **51** followed by the sulfonation, solvation of the complex with tetrabutyl ammonium salt, and its subsequent exchange by Na yield the target compound **IID572** [75] (Scheme 10).

**Scheme 11:**
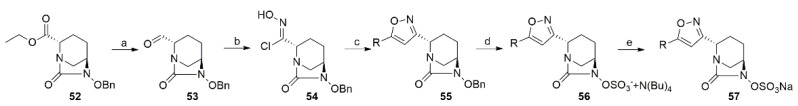
Synthesis of isoxazole derivatives of DBO. Reagents and conditions: (**a**) LiBH_4_, MeOH, −10 to 0 °C, and 4–5 h then 1,3,5-trichloro-1,3,5-triazinane, TEMPO, DCM, 0 °C, and 2 h; (**b**) HONH_2_·HCl, pyridine, EtOH, rt, and 2 h then NCS, pyridine, rt, and 18 h; (**c**) alkyne, TEA, DCM, rt, and 16 h; (**d**) Pd/C, THF, H_2_, and rt then SO_3_-pyridine, pyridine, rt, and 3 h; BuNHSO_4_ and 30 min; (**e**) DOWEX 50WX8 Na^+^ form ion exchange resin and lyophilization.

**Isoxazole derivatives of DBOs:** The C2 position of the DBO scaffold was decorated with a variety of substituted-isoxazole moieties, and the compounds were disclosed by Cubist Pharmaceuticals in 2013 and 2015 in patent applications. The synthesis route can be generalized according to Scheme 11 [76,77]. The synthesis takes the advantage of the commercially available ethyl ester derivative of carboxylic acid **17**. The compound **52** was thus reduced to aldehyde **53** in two steps via an alcohol intermediate that was oxidized to aldehyde by 1,3,5-trichloro-1,3,5-triazinane in the presence of 2,2,6,6-tetramethylpiperidine 1-oxide (TEMPO). The resulting aldehyde undergoes Schiff’s base formation with hydroxylamine hydrochloride, and the intermediate was treated with *N*-chlorosuccinimide to afford the derivative **54**. The compound **54** was then cyclized with an appropriate alkyne to form the isoxazole derivatives **55**, which were converted to the sulfonic salts by standard procedures to obtain the target compounds **57**.

**Scheme 12:**
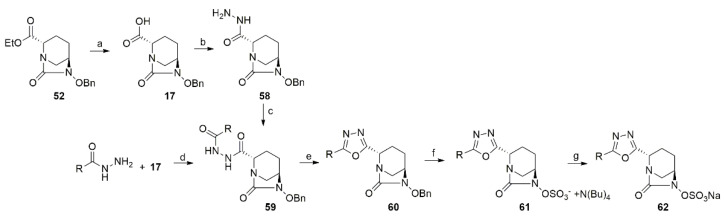
Synthesis of oxadiazole DBO derivatives. Reagents and conditions: (**a**) LiOH, THF/H_2_O, rt, and 16 h; (**b**) BocNHNH_2_, CDI, THF, 0 °C to rt, and 3 h then TFA, DCM, and rt; (**c**) RCOOH, HATU, DIPEA, and DCM; (**d**) HATU, DIPEA, DCM, and rt; (**e**) Tf_2_O, pyridine, DCM, 0 °C to rt, and 3 h; (**f**) Pd/C, THF, H_2_, rt, and 3 h then SO_3_–pyridine, pyridine, rt, and 8 h; BuNHSO_4_, 30 min; (**g**) DOWEX 50WX8 Na^+^ form ion exchange resin and lyophilization.

**Oxadiazole and thiadiazole DBOs:** These compounds were also disclosed by Cubist Pharmaceuticals in 2013 [78]. Two different strategies have been adopted to reach the common intermediate **59**. In one route, the ester hydrolysis of **52** afforded carboxylic acid **17**, which was then reacted with Boc-protected hydrazine to form the derivative **58**. Compound **58** was then coupled with the appropriate organic acids to obtain the derivatives **59**. Alternatively, appropriate hydrazides were coupled with carboxyl group of **17** to afford the compounds **59**. Intermediates **59** were cyclized to oxadiazoles via the activation of carbonyl by triflic anhydride in the presence of pyridine to form compounds **60**. The derivatives were then converted to target compound **62** by following standard procedures (Scheme 12).

**Scheme 13:**
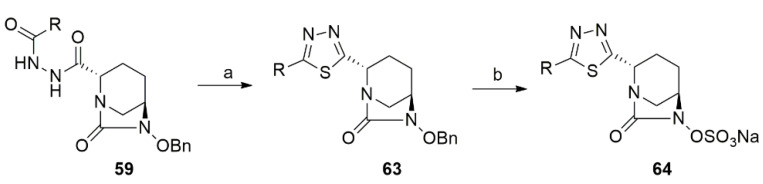
Synthesis of thiadiazole **64**. Reagents and conditions: (**a**) Lawesson’s reagent, THF, 70 °C, and 30 min; (**b**) Pd(OH)_2_/C, THF, H_2_, rt, and 3 h then SO_3_—pyridine, pyridine, rt, 3 h, BuNHSO_4_, 20 min, and DOWEX 50WX8 Na^+^ form ion exchange resin and lyophilization.

The synthesis of the thiadiazole derivatives **64** is depicted in Scheme 13. The thionation of compounds **59** was carried out with Lawesson’s reagent in THF at elevated temperatures, and the resulting intermediates **63** were later converted to the target sulfonic acid salts by benzyl ether hydrogenation, and the sulfonation of the hydroxy derivatives, followed by ion exchange, and lyophilization.

**Scheme 14:**
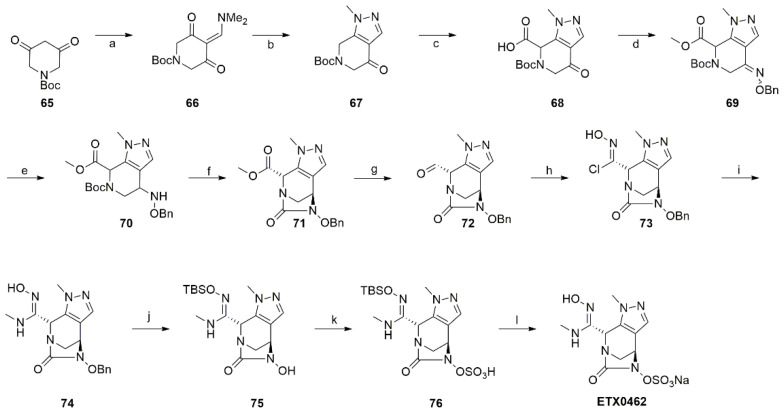
Synthesis of ETX0462. Reagents and conditions: (**a**) DMF-DMA, toluene, 50–80 °C, and 4 h; (**b**) CH_3_NHNH_2_, toluene, EtOH, 25 °C, and 3 h; (**c**) LDA, PMDTA, CO_2_, THF, −78 to 25 °C, and 12 h; (**d**) Me_2_SO_4_, K_2_CO_3_, DMF, rt, and 15 h then O-Benzylhydroxylamine, pyridine, DCM, 20 °C, and 12 h; (**e**) BH_3_/pyridine, HCl/Dioxane, MeOH, 0 to 25 °C, and 14 h; (**f**) triphosgene, TEA, THF, 0 °C to rt, and 12 h; (**g**) LiBH_4_, THF, MeOH, −10 to −5 °C, and 1.5 h then Dess–Martin reagent, DCM, rt, and 1 h; (**h**) NH_2_OH·HCl, pyridine, rt, and 20 min then NCS, DMF, 40 °C to rt, and 16 h; (**i**) NH_2_Me, DCM, rt, and 30 min; (**j**) TBSOTf, 2,6-lutidine, DCM, 30 °C, and 2 h then H_2_, Pd/C, MeOH, EtOAc, rt, and 1 h; (**k**) SO_3_–pyridine, pyridine, rt, and 16 h then HFpyridine, EtOAc, rt, and 2 h; (**l**) DOWEX^®®^50WX8 Na^+^ form ion exchange resin and lyophilization.

**Fused pyrazole DBOs and ETX0462:** The fusion of a pyrazole ring on the carbon scaffold of the DBO ring at C3 and C4 was first disclosed by Novexel and identified **NXL-105** as the lead compound [79]. Entasis therapeutics researchers initiated further optimization based on structure–porin permeation relationships (SPPR), prepared a number of amidine-type pyrazolo DBOs and identified **ETX0462** as the lead for further evaluation [80]. **ETX0462** is the first example where SPPR was successfully incorporated into antibiotic lead optimization, opening another channel for future antibiotic field exploration, and was synthesized in 17 steps [80]. *N*-Boc 3,5-dioxopiperidine (**65**) was heated in toluene with 1,1-dimethoxy-*N,N*-dimethyl-methanamine, and the intermediate was then cyclized with methylhydrazine followed by the installation of carboxylic moiety using LDA and CO_2_ to form compound **68** in three steps. The carbonyl group in **68** was then replaced with O-benzylimine using O-benzylhydroxylamine after the esterification of carboxylic acid part to obtain compound **69**. Next, the imine in **69** was reduced with borane followed by cyclization with triphosgene to form the urea intermediate **71**. The reduction of the ester part in the urea intermediate to alcohol furnishes aldehyde **72** in two consecutive steps upon oxidization. The following step was the conversion of aldehyde to hydroxylamine by reacting **72** with hydroxylamine hydrochloride, and the intermediate imine was then reacted with *N*-chlorosuccinimide (NCS) to form the chloro compound **73** in two steps. The chloro group was then substituted with methylamine to form the intermediate compound **74**, which was debenzylated after protecting the OH group with TBS to form the hydroxy derivative **75**. The hydroxy intermediate was then converted to the final product **ETX0462** after sulfation, the deprotection of TBS, and ion exchange [80] (Scheme 14).

**ETX0462** is analogous to the **NXL-105** developed by Novexel and possesses antibacterial properties in addition to lactamase inhibition strength [79]. **ETX0462** has potent in vitro and in vivo activity against *Pseudomonas aeruginosa, Stenotrophomonas maltophilia, Acinetobacter baumannii, Burkholderia cepacia,* and *Klebsiella pneumoniae*. The compound shows an antibacterial profile by inhibiting penicillin-binding proteins (PBPs) as well as various BLAs produced by numerous Gram-negative pathogens [81].

**Scheme 15:**
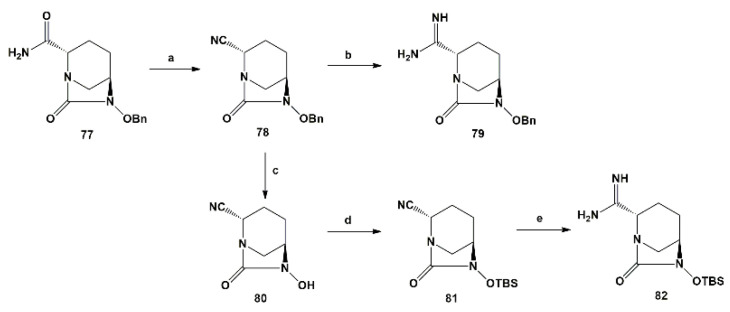
Synthesis of key amidine intermediates **79** and **82**. Reagents and conditions: (**a**) trifluoroacetic anhydride, CH_2_Cl_2_, 0 °C-rt, and 3 h; (**b**) Al(Me)_3_, NH_4_Cl, CH_2_Cl_2,_ 0 °C-rt, and 16 h; (**c**) Pd/C (wet), EtOAc/CH_2_Cl_2_, H_2,_ 45 psi, rt, and 2 h; (**d**) TBSCl, imidazole, CH_2_Cl_2_, rt, and 16 h; (**e**) Al(Me)_3_, NH_4_Cl, CH_2_Cl_2,_ 0 °C-rt, and 40 h.

**Amidine substituted DBOs:** Our group synthesized the amidine derivative of avibactam by replacing its amide group at the C2 of the DBO ring. The amidine-substituted derivative was further modified by reacting the NH_2_ group of the amidine moiety with numerous functionalities [82,83,84,85,86,87]. Amidine derivatives **79** and **82** were the key intermediates for the synthesis of the target compounds. The synthesis of these compounds is shown in Scheme 15. The commercially available amide **77** was dehydrated to cyano compound **78** via a slight modification to the known procedure [88]. The conversion of the cyano group in **78** was achieved with a 64% yield via Garigipati’s reaction [89,90] using trimethylaluminum and ammonium chloride in DCM.

The synthesis of intermediate **82** started from **78** via the palladium-catalyzed hydrogenation of the benzyl ether using EtOAc/CH_2_Cl_2_ as a solvent mixture leading to hydroxy compound **80** [88]. The hydroxyl group in **80** was then protected by TBS using *tert*-butyldimethylsilyl chloride in the presence of imidazole, and the resulting compound **81** was subjected to the aforementioned Garigipati conditions to form the target compound **82** (Scheme 15) [82,83].

**Scheme 16:**
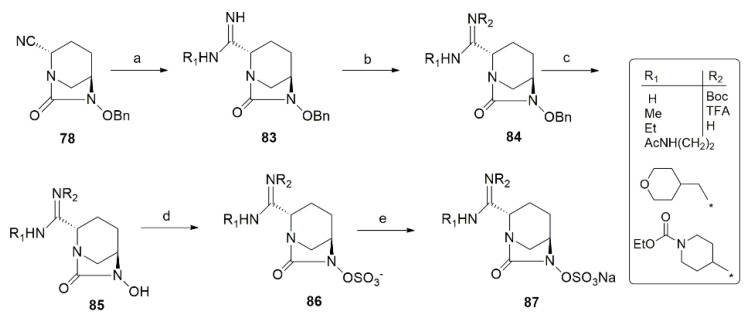
Synthesis of amidine derivatives **87**. Reagents and conditions: (**a**) Al(Me)_3_ or TMSOTf, NH_4_Cl or R_1_NH_2_, 0 °C—rt, and 16 h; (**b**) (Boc)_2_O, TEA, DCM, rt, and 48 h or triflouroacetic anhydride, TEA, DCM, 0 °C—rt, and 4 h; (**c**) Pd/C (wet), MeOH or EtOAc, H_2_, 16 h, and rt; (**d**) SO_3_–pyridine, pyridine or SO_3_—NMe_3_, TEA/MeOH, rt, and 16 h; (**e**) TFA, DCM, 0 °C, and 3.5 h; Dowex-50wx Na^+^.

A series of amidine derivatives (Scheme 16) was synthesized from the direct reaction of R-NH_2_ with the cyano compound **78** in the presence of either trimethylaluminum or trimethylsilyltriflate in moderate yields. The debenzylation of the thus formed intermediates was either unsuccessful or led to the cumbersome purification of the hydroxy compounds. Therefore, the NH moiety was first protected by Boc or TFA, and the resulting compounds were hydrogenated using standard conditions with palladium catalyst to form the compounds **85**. The hydroxy derivatives were then reacted with SO_3,_ leading to the final target compounds **87** after ion exchange and lyophilization [85].

**Scheme 17:**
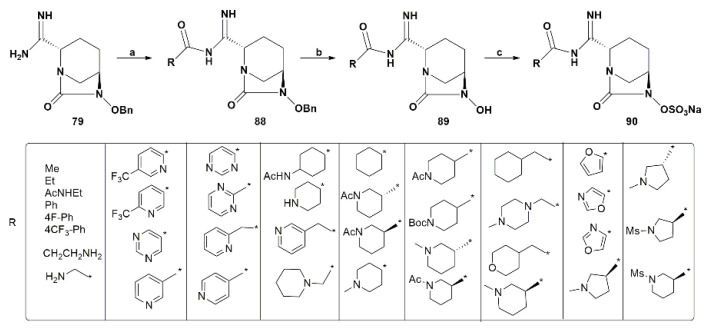
Synthesis of amidine-substituted compounds. Reagents and conditions: (**a**) HATU/DCC, DMAP/DIPEA, DMF/CH_2_Cl_2_, rt, and 16–24 h; (**b**) Pd/C (wet), THF/EtOAc, TEA, H_2,_ rt, and 16 h; (**c**) SO_3_—pyridine or SO_3_—NMe_3_, TEA, THF/water, rt, and 16 h; Dowex-50wx Na^+^.

Another series of amidine derivatives (Scheme 17) was synthesized from the direct reaction of R-COOH with amidine **79** under coupling conditions in moderate yields. The resulting amides **88** were hydrogenated using palladium catalyst to form compounds **89**. The hydroxy derivatives were then reacted with SO_3_ to obtain the final target compounds **90** after ion exchange and lyophilization [85].

**Scheme 18:**
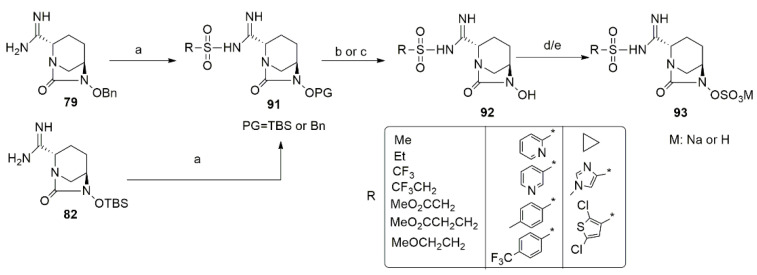
Synthesis of sulfonylamidine DBO derivatives. Reagents and conditions: (**a**) RSO_2_Cl or (RSO_2_)_2_O, TEA, CH_2_Cl_2_ or EtOAc, 0 °C-rt, and 16 h; (**b**) H_2_, Pd/C, and THF or EtOAc or MeOH, rt, and 2–18 h; (**c**) TBAF, THF, 0 °C- rt, and 1 h; (**d**) SO_3_–pyridine, pyridine, rt, and 16 h; (**e**) Dowex-50wx Na^+^ or preparative HPLC.

One more series of sulfonylamidine-substituted DBOs was synthesized from either amidine **79** or **82** according to Scheme 18. The NH_2_ of the amidine was first reacted with a variety of sulfonyl chlorides or sulfonic anhydrides, leading to the formation of respective intermediate derivatives **91**. The individual derivatives were then subjected to the removal of protecting groups (TBS or Bn) to obtain the hydroxy derivatives **92**. The following steps for sulfation and preparative HPLC or ion exchange chromatography led to the formation of the required sodium salts **93** [86].

**Scheme 19:**
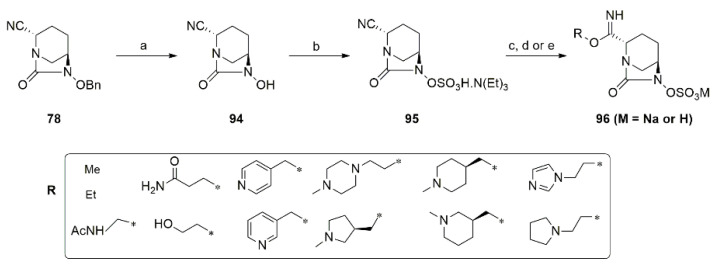
Synthesis of imidate-substituted DBO derivatives. Reagents and conditions: (**a**) Pd/C, EtOAc, H_2_, rt, and 4 h; (**b**) SO_3_-NMe_3_, TEA, THF/H_2_O, rt, and 16 h; (**c**) MeOH, MeONa, 0 °C, and 20 min; (**d**) EtOH, EtONa, rt, and 15 h; (**e**) RH, NaH, and DMF or THF/DCM, rt, 30 min, and Dowex-50wx Na^+^ or preparative HPLC.

**Imidate-substituted DBOs:** Our group recently reported a series of DBO derivatives in which the C2 position of the ring was decorated with various imidate substituents. Synthesis was accomplished according to Scheme 19. At first, the cyano compound **78** was hydrogenated using palladium catalysis followed by the reaction of the intermediate hydroxy compound **94** with SO_3_–TEA complex to form the sulfonic salt **95**, which was then converted to the imidates by reacting it with appropriate RONa or alcohols in the presence of NaH in DMF or using THF/DCM as a solvent in low yields. The final compounds were acquired by the preparative HPLC purification of the intermediates or by passing the intermediates through a column filled with Dowex-50wx Na^+^ ion exchange resin to form the respective sodium salts.

**Boronic acids β-lactamase inhibitors:** The interaction of boronic acids and their esters via covalent binding to the active site serine residue of certain proteases [91,92] provided the basis for their exploration as BLIs. The first report on the inhibition of the penicillinase by phenylboronic acid published by the Waley group in 1978 [93] led them to exploring of various boronic acids, such as BLIs, in the 1980s. In the following years, other researchers identified more potent boronic acid inhibitors of AmpC in the nano molar range [94]. It was later determined that the interaction between the boron atom of the inhibitor and the serine residue of BLA proceeds through a covalent tetrahedral adduct that mimics the acylation or deacylation step of the BLA-mediated hydrolysis of substrate [95]. The exploration of boronic acids has now become an active field of research for the identification of more potent BLIs, especially for MBLs [96].

**Vaborbactam:** Vaborbactam, formerly **RPX7009**, the first FDA-approved cyclic boronic acid BLI [92], has shown the ability to inhibit ESBLs, class C BLAs, Class A carbapenemases (KPCs) [97,98,99,100], and some class D BLAs [94,100]. In addition, recent studies show that vaborbactam is also a weak inhibitor of class B MBLs [100,101]. Vaborbactam is a cyclic boronic acid inhibitor [100] that exhibits high selectivity towards serine BLs compared to the serine proteases in silico [94]. The design of vaborbactam and analogous compounds was based on the structures described by Ness et al. [102]. The authors conducted an in silico docking investigation of several potential structures with the selected class A, C, and D BLs, which led them to the identification of the core structure of vaborbactam [32].

**Scheme 20:**
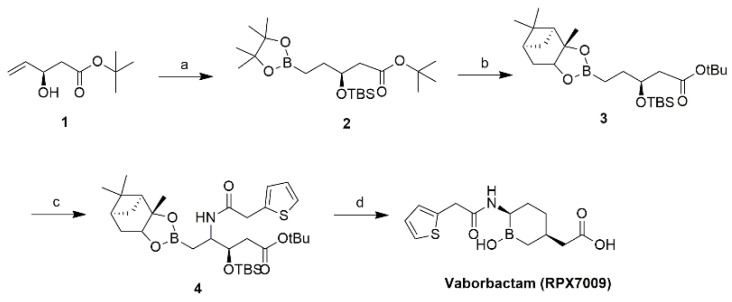
Synthesis of vaborbactam. Reagents and conditions: (**a**) TBSCl, imidazole, DCM, rt, and 2 h then [Ir(COD)Cl]_2_, dppb, pinacolborane, rt, and 4 h; (**b**) (+)-pinanediol, rt, and 24 h; (**c**) n-BuLi, THF, −90 °C, and 1h; LiHMDS, THF, −78 °C-rt, and 16 h; 2-(thiophen-2-yl)acetic acid, EDCl, HOBt, NMM, DCM, 0 °C to rt, and 30 min (**d**) 3 N HCl, dioxane, reflux, and 1.5 h.

The synthesis of vaborbactam (Scheme 20) and 20 analogous compounds described by Hecker et al. can be completed in six steps and can achieve an overall yield of 30% [32]. The starting material, *(R)-tert*-butyl 3-hydroxypent-4-enoate **(1)**, was purified from its racemate by means of lipase mediated kinetic resolution. The pure enantiomer **1** was protected with TBS and was subsequently converted to pinacol boronate **2** by regioselective hydroboration catalyzed by [Ir(COD)Cl]_2_. The stable boronate **3** was obtained by reacting **2** with pinanediol at room temperature. The compound **3** was then converted to a chloro intermediate by Matteson’s protocol at −90 °C followed by the stereospecific displacement of chloro by hexamethyldisilazide, and the subsequent in situ acylation thus led to the formation of acylamidoboronate **4**. The removal of protecting groups and cyclization in refluxing aqueous HCl afforded the cyclic boronate (vaborbactam) as a white solid with 64% yield after purification.

**Scheme 21:**
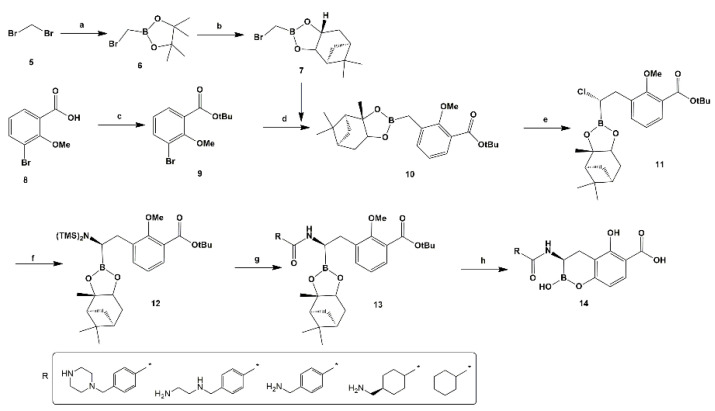
Synthesis of cyclic boronic acids. Reagents and conditions: (**a**) n-BuLi, B(O^i^Pr)_3_, THF, −100 °C to rt, and 5 h then CH_3_SO_3_H, −0 °C, and 1 h; pinacol, rt, and 16 h; (**b**) (+)-pinanediol, THF, rt, and 16 h; (**c**) oxalyl chloride, cat. DMF, DCM, rt, and 14 h then t-BuOH, 70 °C, and 16 h; (**d**) n-BuLi, THF, −100 °C, and 3 h; (**e**) LDA, DCM, THF, −100 °C, and 30 min; ZnCl_2_, THF, −100 °C to rt, 16 h; (**f**) LiHMDS, THF, −100 °C to rt, and 16 h; (**g**) MeOH, THF, rt, and 2 h; RCOOH, HATU, TEA, −20 °C to rt, and 16 h; (**h**) BBr_3_, DCM, −78 °C, and 3 h.

**Cyclic boronates:** The SBL/PBP inhibition ability of boronic acids and the clinical trial phase of vaborbactam prompted the Novartis research team towards the synthesis of cyclic boronic acids. Thus, several derivatives with a variety of R-groups targeting the inhibition of MBLs were disclosed in a patent application [103]. Of these cyclic boronates, the selected candidates were resynthesized in addition to two new compounds that were designed on the basis of molecular modeling. The compounds (Scheme 21) manifested the potent inhibition of MBL and NDM-1 [104].

**Taniborbactam:** Taniborbactam (**VNRX-5133**) [105] is a cyclic boronic acid derivative that is now in clinical trials. It has been identified as a pan spectrum inhibitor of both SBLs and MBLs. It restores the activity of BL antibiotics against carbapenem-resistant *Pseudomonas aeruginosa*, *Enterobacteriaceae* [106], and *Klebsiella pneumoniae* [107]; ESBLs; and class D OXA enzymes with carbapenem-hydrolyzing capability [108]. Taniborbactam in combination with cefepime has demonstrated therapeutic potential against carbapenemase-producing *Enterobacterales* and carbapenem-resistant *Pseudomonas* isolates in addition to MBLs such as NDM-1, and VIM-1/2 [109,110]. Recently, a combination of cefepime with taniborbactam, which is currently in the clinical development phase, has qualified the safety and pharmacokinetic investigations at single and multiple doses in human subjects [111].

**Scheme 22:**
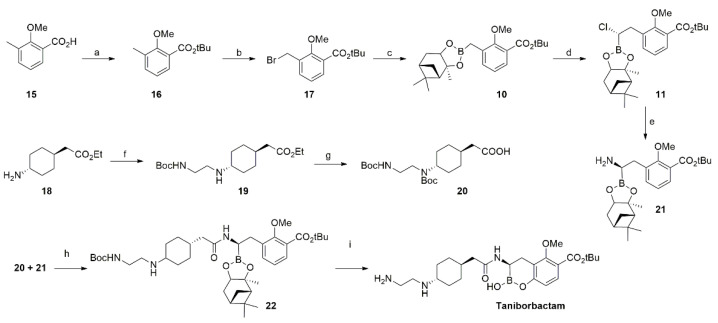
Synthesis of Taniborbactam. Reagents and conditions: (**a**) oxalyl chloride, cat. DMF, CH_2_Cl_2_, rt, and 90 min then 2-methylpropan-2-ol, 40 °C, and 18 h; (**b**) *N*-bromosuccinimide, benzoyl peroxide, CCl_4_, reflux, UV, and 5 h; (**c**) bis[(+)-pinanediolato]diboron, Pd(dppf)Cl_2_, KOAc, 1,4-dioxane, 95 °C, and 16 h; (**d**) CH_2_Cl_2_, THF, *n*-BuLi, −100 °C, and 45 min and then 10 in THF, ZnCl_2_, −95 °C to rt, and overnight; (**e**) lithium bis(trimethylsilyl)amide, THF, −100 °C to −78 °C, and 2 h then MeOH, THF, −10 °C to rt, and 1 h; (**f**) K_2_CO_3_, CH_2_Cl_2_/H_2_O (1:1), rt, and 1 h then 2-(boc-amino)ethyl bromide, benzyltriethylammonium chloride, reflux, and 18 h; (**g**) di-*tert*-butyl dicarbonate, *N,N*-diisopropylethylamine, reflux, 16 h, LiOH·H_2_O, THF/EtOH/H_2_O (1:2:1), rt, and 5 h; (**h**) 20, triethylamine, PyBOP and then crude 21, rt, and 75 min; (**i**) BCl_3_, CH_2_Cl_2_, −78 °C, and 1 h.

The synthesis of taniborbactam and its analogous derivatives was disclosed in an early patent [103,112]. In the following years, the synthesis routes were modified and published in 2019. According to this report, taniborbactam was synthesized (Scheme 22) in 11 steps from ester **16**, which was prepared via Matteson homologation from 2-methoxybenzoic acid **15** after the carboxyl group esterification. The bromination of the methyl group followed by hydroboration with (+)-pinanediol afforded the requisite precursor **10**. The boronate **10** was then subjected to homologation, which was accomplished by in situ-generated dichloromethyllithium, to provide a moderate yield of the (*S*) enantiomer of chloro-derivative **11**. The chloro in **11** was converted to amine by treating **11** with LiHMDS followed by reacting the intermediate with anhydrous methanol to obtain amino compound **21** with an inverted configuration.

The required carboxylic acid **20** was prepared from commercially available ethyl 2-(trans-4-aminocyclohexyl) acetate hydrochloride (**18**) via *N*-alkylation in biphasic conditions in the presence of benzyltriethylammonium chloride to furnish **19** in 49% yield. The final compound **20** was obtained by Boc protection of NH_2_ in **19** followed by saponification. Next, the coupling of compounds **20** and **21** was achieved by using (benzotriazole-1-yloxy)tripyrrolidinophosphonium hexafluorophosphate (PyBOP) as a coupling reagent in the presence of triethylamine in 34% yield. The intermediate amide was then cleaved for the protecting groups in one pot by using BCl_3_ at low temperatures, leading to the formation of taniborbactam, which was purified by preparative HPLC [110].

**Scheme 23:**
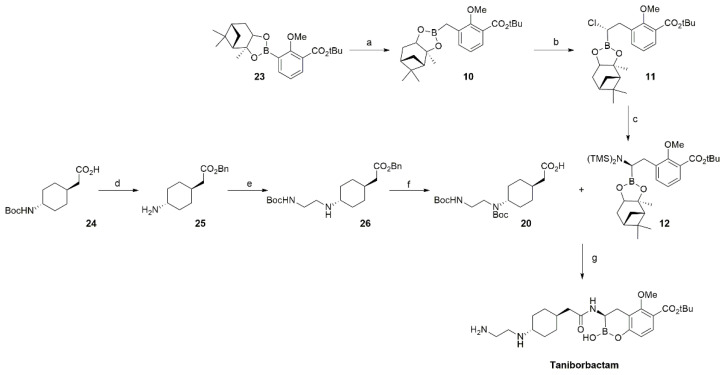
Modified synthesis of Taniborbactam. Reagents and conditions: (**a**) ClCH_2_I, n-BuLi, −100 °C to rt, and 17.5 h; (**b**) n-BuLi, DCM; ZnCl_2_, THF, −100 °C to rt, and 16 h; (**c**) LiHMDS, THF, −20 °C, 17 h (**d**) K_2_CO_3_, DMF, and BnBr then 4N HCl and dioxane; (**e**) BocNHCH_2_CH_2_CHO, NaBH(OAc)_3_, DCE (**f**) Boc_2_O, TEA, H_2_, and Pd/C; (**g**) HATU, DMA, NMM, rt, 2.5 h, and BCl_3_; DCM, −78 °C to rt, and 2 h.

Recently, further amendments in the synthesis of chloro-compound **11** have been described, as shown in Scheme 23. The commercially available compound 3-boromo-2-methoxybenzoic acid was converted to compound **23** in two steps: first, esterification by isobutylene, followed by a reaction with (+)-pinanediol. Compound **23** was reacted with chloromethyl lithium, generated by reacting n-BuLi with chloroiodomethane in situ, to yield compound **10** in an excellent yield. Secondary homologation was achieved by the anion that was generated in situ from n-BuLi in DCM to form the stereoselective *(S)*- α-chloro-boronate **11**. The displacement of the chloro group in **11** with lithium bis(trimethylsilyl) amide afforded the intermediate α-silylaminoboronate **12**, which was directly combined with carboxylic acid [110] **20** using hexafluorophosphateazabenzotriazoletetramethyl uronium (HATU) and *N*-methylmorpholine (NMM), affording the resultant amide. The amide intermediate was then deprotected and cyclized to form the final target compound, taniborbactam [106] (Scheme 23).

**QPX7728:** It is another bicyclic boronic acid derivative that inhibits class A ESBLs and carbapenemases such as KPC, as well as P99 from class C. It is a promising inhibitor of class D carbapenemases, such as OXA-48 from *Enterobacteriaceae* and OXA-23/24/58 from *Acinetobacter baumannii,* as well as MBLs such as NDM-1, VIM-1 and IMP-1 [113].

**Scheme 24:**
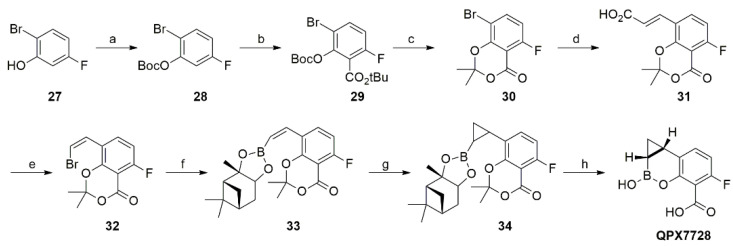
Synthesis of QPX7728. Reagents and conditions: (**a**) Boc_2_O, DMAP, DCM, and rt; (**b**) LDA, THF, Boc_2_O, DMAP, DCM, and rt; (**c**) TFA, acetone, TFA, TFAA, and 65 °C; (**d**) acrylic acid, Pd(OAc)_2_, P(*o*-toly)_3_, Et_3_N, DMF, and 100 °C; (**e**) Br_2_, CHCl_3_, 0 °C, Et_3_N, DMF, and 0 °C; (**f**) B_2_((+)pinanediol)_2_, PdCl_2_(dppf), KOAc, dioxane, and 60 °C; (**g**) CH_2_N_2_, Pd(OAc)_2_, THF, and −10 °C to rt; (**h**) 3 N NaOH, dioxane, and rt, then TFA, TES, and *i*-BuB(OH)_2_.

The synthetic procedures for **QPX7728** are described in Scheme 24. The hydroxyl group in commercially available 2-bromo-5-fluorophenol was Boc-protected to produce compound **28**, which, upon treatment with lithium diisopropylamide (LDA) followed by acyl transfer, produced the compound **29**. Next, Boc protections were replaced with single acetonide protection, and the resulting compound **30** was coupled with acrylic acid under Heck reaction conditions to produce compound **31**. The bromination of compound **31** followed by decarboxylation afforded cis-vinylbromide compound **32**, which was converted to vinylboronate **33** by means of palladium-catalyzed borylation with (+)-pinanediol. The subsequent cyclopropanation of the compound **33** with diazomethane resulted in a mixture of diastereomers, which was separated by column chromatography followed by the deprotection of the acetal pinandiol protections, resulting in **QPX7728** being the stereoisomer [114].

**Scheme 25:**
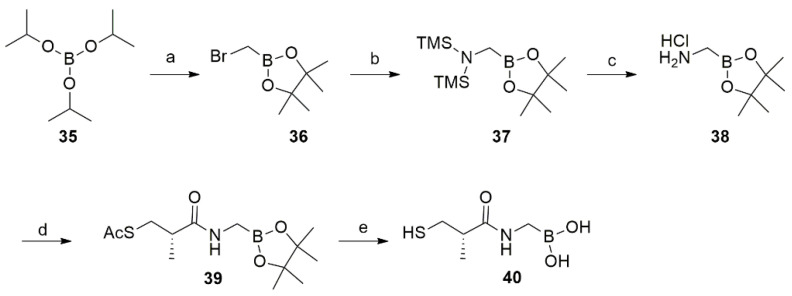
Synthesis of acyclic boronic acids. Reagents and conditions: (**a**) CH_2_Br_2_, n-BuLi, THF, −78 °C, and 2 h; CH_3_SO_2_OH, 0 °C, and 1 h; pinacol, 0 °C, and 1 h; (**b**) LiHMDS, THF, −78 °C to rt, and overnight; (**c**) HCl/1,4- dioxane, Et_2_O, −60 °C to rt, and 3 h; (**d**) 3-(prop-1-en-2-ylthio)propanoic acid, TBTU, DIPEA, DMF, −10 °C, and 2 h; (**e**) 2-methylpropylboronic acid, HCl (aq), CH_3_OH/hexane (1:1), rt, and overnight.

**Scheme 26:**
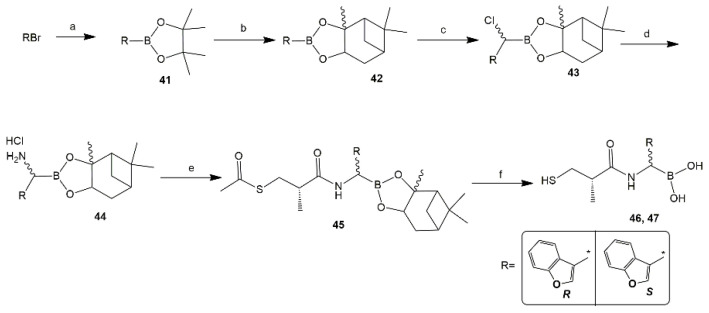
Synthesis of acyclic boronic acids. Reagents and conditions: (**a**) Nis(pinacolato)diboron, CuBr, NaOtBu, DMF, Ar, 80 °C, and 10 h; (**b**) (+)-pinanediol or (−)-pinanediol, Et_2_O, rt, and overnight; (**c**) CH_2_Cl_2_, n-BuLi, ZnCl_2_, THF, −100 °C to rt, and overnight; (**d**) LiHMDS, THF, −78 °C to overnight, HCl/1,4-dioxane, Et_2_O, −60 °C to rt, and 3 h; (**e**) 3-(prop-1-en-2-ylthio)propanoic acid, TBTU, DIPEA, DMF, −10 °C, and 2 h; (**f**) 2-methylpropylboronic acid, HCl (aq), CH_3_OH/hexane (1:1), rt, and overnight.

**Acyclic boronic acids:** Recently, Li et al. reported that several derivatives of (2′S)-(1-(3′-mercapto-2′-methylpropanamido)methyl)boronic acid (MMB) are dual inhibitors of SBL and MBL. The core structure of MMB, which is designed using a pharmacophore fusion strategy, manifested the inhibition of VIM-2 and KPC-2. Further structural optimization led to the synthesis (Scheme 25 and Scheme 26) of structurally diverse analogs of the core structure MMB, and selected candidates, **40**, **46**, and **47**, restored the efficacy of meropenem against NDM-1, KPC-2, or AmpC, producing clinical isolates of *E. coli* and *K. pneumoniae* [115].

**Miscellaneous MBL inhibitors:** Metallo β-lactamases are structurally different from the serine β-lactamases, and hence, the BL hydrolysis mechanism also varies. The hydrolysis action in MBLs mainly depends on the presence of the Zn ions. The metal ions participate as the Lewis acid during water molecule activation, which, in turn, affords the hydroxide ions to attack the carbonyl function of the BL. Therefore, MBL inhibition is often focused on the binding of Zn ions. Several structurally diverse classes such as dicarboxylic acids [116], triazoles [116], phosphonates, boronates, and thiol-based MBL inhibitors, [23] have been synthesized and explored to determine their enzyme inhibition activity, and the literature describing these classes has been extensively reviewed recently [117]. We will focus on the newly emerging classes of compounds that have not yet been reviewed.

**Scheme 27:**
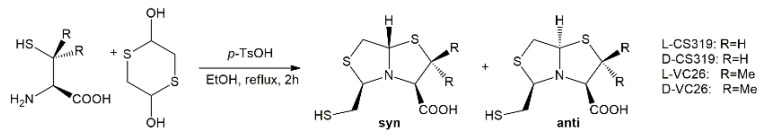
Bisthiazolidine synthesis.

**Bisthiazolidines:** This class of compounds was described by the Spencer group. Four novel bisthiazolidine (BTZ) (Scheme 27) compounds were prepared and showed the inactivation of NDM-1, VIM-2, and VIM-24 MBLs in vitro. In addition, these structures also restored the activity of imipenem against *Klebsiella pneumoniae*- and *Pseudomonas aeruginosa*-producing VIM-24 and VIM-2, respectively, and the NDM-1-producing *Escherichia coli* and *Acinetobacter baumannii* [118,119,120]. The compounds were prepared in one step, starting with D- or L-cysteine (or D/L-penicillamine) and reacting it with 1,4-dithiane-2,5-dithiol in refluxing ethanol in the presence of p-toluenesulfonic acid. The compounds were produced at high yields (as *syn* and *anti* conformers) [118,119,120].

**Scheme 28:**
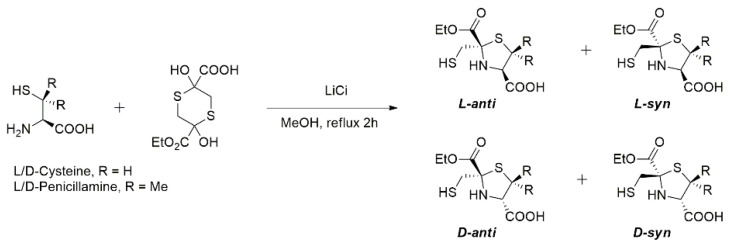
2-mercaptomethyl-thiazolidine synthesis.

**2-Mercaptomethyl-thiazolidines:** The Spencer group further described 2-mercaptomethyl-thiazolidines (MMTZs) (Scheme 28) as potent competitive inhibitors of B1 MBLs in vitro. MMTZs penetrate multiple *Enterobacterales* and restore carbapenem potency against clinical isolates expressing B1 MBLs [121]. The compounds were synthesized by the condensation of L- or D- cysteines (or L/D-penicillamine) with diethyl 2,5-dihydroxy-1,4-dithiane-2,5-dicarboxylate in refluxing methanol. Syn/anti diastereomeric mixtures that were produced could be easily purified, and evaluated as pure compounds [122].

**Scheme 29:**
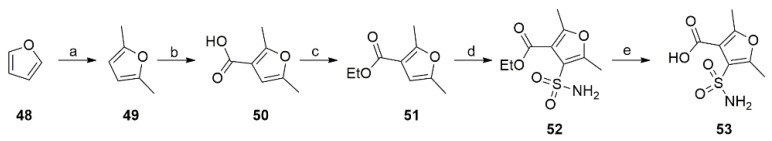
Synthesis of 2,5-dimethyl-4-sulfamoylfuran-3-carboxylic acid (**53**). Reagents and conditions: (**a**) CH_3_Br, TMEDA, n-BuLi/hexane, 0 °C, THF, rt-reflux, and 1 h; (**b**) Me_2_AlCl, CO_2_, toluene, rt, 16 h; (**c**) HCl, EtOH, rt, and 16 h; (**d**) ClSO_3_H, acetonitrile, NH_4_OH, 0 °C, and 3 h; (**e**) NaOH, MeOH/H_2_O, rt, and 16 h.

**Scheme 30:**
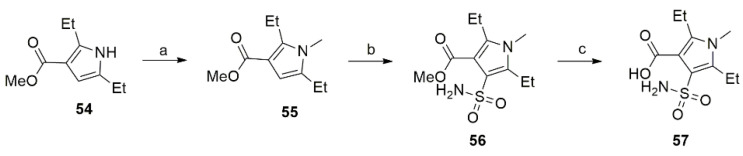
Synthesis of compound **57**. Reagents and conditions: (**a**) bromomethane, DBU, acetonitrile, 80 °C, and 16 h; (**b**) ClSO_3_H, acetonitrile, NH_4_OH, 0 °C, and 5 h; (**c**) NaOH, MeOH/H_2_O, rt, and 16 h.

**Sulfamoyl heteroarylcarboxylic acids:** Sulfamoyl heteroarylcarboxylic acids (SHCs) have been described as competitive inhibitors of MBLs. Several SHC derivatives have been synthesized according to Scheme 29 and subjected to MBL inhibition tests in vitro. Compound **53** manifested the satisfactory inhibition of MP-1-, VIM-1-, and NDM-1-producing *Enterobacteriaceae* in combination with meropenem both in vitro and in vivo. Replacing heterocyclic oxygen with nitrogen, a series of compounds was synthesized, of which compound **57** (Scheme 30) proved to be a potent inhibitor of IMP-1, NDM-1, and VIM-2 in vitro as well as in vivo [123].

**Scheme 31:**
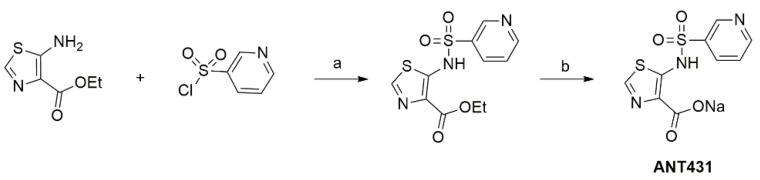
Synthesis of **ANT431**. Reagents and conditions: (**a**) NaH, THF, 0 °C to rt, and 4 h; (**b**) LiOH, THF/H_2_O, rt, and 48 h then THF, 0.1 M NaOH, sonication, 10 min, and H_2_O.

**ANT431:** Owing to the weak MBL inhibition capability of pyridine-2-carboxylic acid, several derivatives were synthesized and studied to determine the structure–activity relationship (SAR) in combination with imipenem against clinical isolates of *Escherichia coli* and *Klebsiella pneumoniae* harboring NDM-1 [124]. The SAR studies identified **ANT431** (Scheme 31) as the potential molecule for further preclinical investigations. Further enzymatic assays showed that **ANT431** can potentiate the efficacy of meropenem against NDM-1-producing *Escherichiacoli* in a murine thigh infection model at a 28 μM concentration [125].

**Scheme 32:**
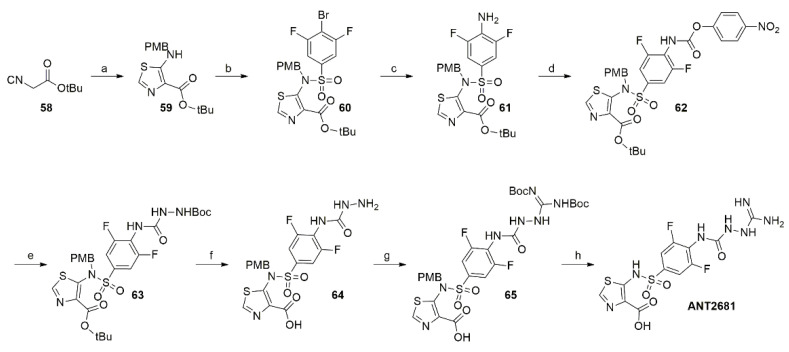
Synthesis of **ANT2681**. Reagents and conditions: (**a**) 4-methoxybenzyl isothiocyanate, *t*BuOK, THF, rt, and 2 h; (**b**) 4-bromo-3,5-difluoro-benzenesulfonyl chloride, NaH, THF, 0 °C, and 1.5 h; (**c**) NH_3_, K_3_PO_4_, Xantphos, Pd_2_(dba)_3_, dioxane, 100 °C, and 5 h; (**d**) (4-nitrophenyl) chloroformate, toluene, reflux, and 3 h; (**e**) *tert*-butyl carbazate, DIPEA, THF, 0 °C to rt, and 3 h; (**f**) HCl, Et_2_O, rt, and 24 h; (**g**) DIPEA, *N*,*N*′-bis(*tert*-butoxycarbonyl)-1*H*-pyrazole-1-carboxamidine, DMF, rt, and 5 h; (**h**) TFA, rt, and 3 h.

**ANT2681:** The structure of **ANT431** was further optimized to increase its inhibition activity and physicochemical properties, and the same research group prepared a diverse variety of compounds that culminated with the identification of **ANT2681** (Scheme 32), which was determined to be a promising NDM inhibitor against a wide variety of clinical strains [126]. In preclinical investigations, **ANT2681** showed competitive inhibition against 1687 MBL-positive strains of *Enterobacterales* in combination with meropenem [127].

**Scheme 33:**
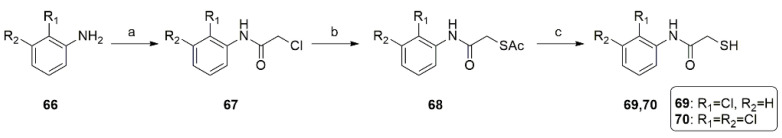
Synthesis of *N*-mercaptoacetamides **69** and **70**. Reagents and conditions: (**a**) 2-chloroacetyl chloride, DIPEA, DCM, and 0 °C; (**b**) Pot. thioacetate, acetone, and rt; (**c**) KOH, MeOH/H_2_O, and rt.

***N*-Aryl mercaptoalkylamides:** A series of *N*-aryl mercaptoacetamide derivatives was synthesized according to Scheme 33, and the compounds **69** and **70** showed potential for the inhibition of NDM-1 in *Klebsiella pneumoniae* isolates in combination with imipenem [128]. Recently, another series of *N*-aryl mercaptopropionamide has been reported to contain a hit structure that is capable of reducing the MIC value of accompanying imipenem against B1 MBLs expressing *Escherichia coli* strains up to 256-fold [129].

**Scheme 34:**
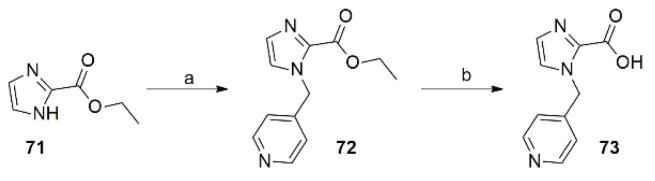
Synthesis of compounds 1*H*-imidazole-2-carboxylic acid **73**. Reagents and conditions: (**a**) RX, NaH, THF, rt, and 2 h; (**b**) NaOH, EtOH/H_2_O, rt, and 1 h.

**1*H*-imidazole-2-carboxylic acids:** A library of 1*H*-imidazole-2-carboxylic acids was prepared following the analogous conditions depicted in Scheme 34. Compound **73** exhibited the potent inhibition of the VIM-2 MBL expressed in *Escherichia coli* in combination with meropenem [130].

**Scheme 35:**
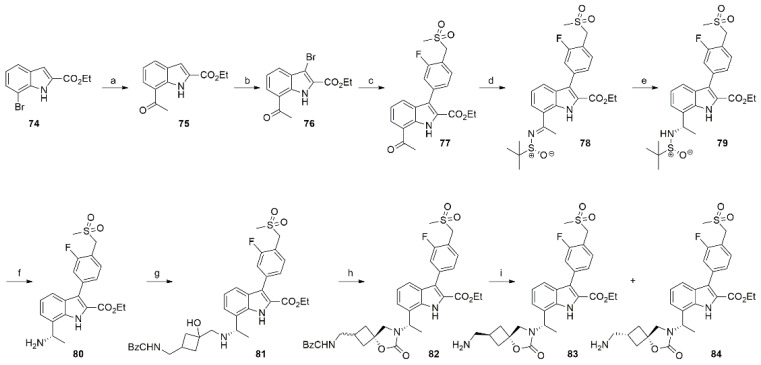
Synthesis of compounds indole-2-carboxylate **83**. Reagents and conditions: (**a**) 1-(vinyloxy)butane, Et_3_N, Pd(OAc)_2_/dppp, EtOH, 90 °C, and 18 h; (**b**) NBS, MeCN, and 0 °C to rt; (**c**) 2-(3-Fluoro-4-((methylsulfonyl)methyl)phenyl)-4,4,5,5-tetramethyl-1,3,2-dioxaborolane, Pd(dppf), DCM, Na_2_CO_3_, 1,4-dioxane, 80 °C, and 1 h; (**d**) (*R*)-*t*Bu-sulfinimide, Ti(OEt)_4_, THF, 75 °C, and 22 h; (**e**) L-Selectride, THF, 50 °C, and 3 h; (**f**) 4 M HCl, THF/EtOH, rt, and 2 h; (**g**) benzyl (1-oxaspiro [2.3]hex-5-ylmethyl)carbamate, LiClO_4_, toluene, 110 °C, and 20 h; (**h**) 1,1′-carbonyldiimidazole (CDI), MeCN, rt, 44 h, Pd/C, H_2_, AcOH, rt, and 1 h then (**i**) LiOH, THF, H_2_O, 0 °C to rt, and 20 h.

**Indole-2-carboxylates (InCs):** Designed based on the high-throughput screening of the NDM-inhibitors, indole 2-carboxylates were identified as BLA-stable BL mimics. Consequent to the screening results, a number of indole-2-carboxyl derivatives were synthesized [131] and studied for their in vitro as well as in vivo efficacy against MBLs (NDM-1, VIM-1, VIM-2, and IMP-1). The initial structure–activity relationship investigations revealed that compound **83** (Scheme 35) was a potential MBL inhibitor. The compound was then assessed in combination with meropenem and doripenem against several *Enterobacterales* in vitro. The results showed that these combinations possess excellent inhibition activity with average MIC_90_ value of 1-2 mg/L. The highest activity was observed against *Citrobacter* spp., *Serratia marcescens,* and *Proteus mirabilis* in combination with meropenem (MIC_90_ 0.125–0.5 mg/L). The combination of **83** with meropenem showed activity that was up to six-fold higher compared to taniborbactam–meropenem. Additionally, in vivo studies conducted in mice models infected with carbapenem-resistant strains revealed that the combination of **83**–meropenem has substantial potential for clinical trials [131].

**Scheme 36:**
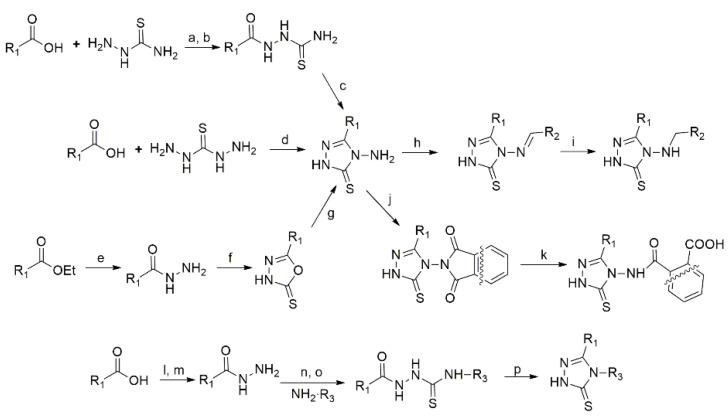
Synthesis of derivatives from compounds 1,2,4-triazole-3-thione. Reagents and conditions: (**a**) SOCl_2_, toluene, and reflux; (**b**) pyridine, toluene, and rt; (**c**) aqueous NaOH and reflux; (**d**) neat and 160 °C; (**e**) hydrazine hydrate, neat, 120 °C, and sealed tube; (**f**) CS_2_, KOH, EtOH, and reflux; (**g**) hydrazine hydrate, EtOH, 100 °C, and sealed tube; (**h**) AcOH, reflux or 35% HCl (cat.), EtOH, refl., and 4–12 h; (**i**) NaBH_4_, EtOH, 0 °C to r.t. 6–24 h or NaBH_4_, MeOH, and reflux; (**j**) pyridine and reflux; (**k**) KOH, EtOH/H_2_O, and rt; (**l**) EtOH, H_2_SO_4_, reflux, and 5 h; (**m**) EtOH, hydrazine hydrate, 100 °C, and sealed tube; (**n**) DPT, DMF, sealed tube, 55 °C, and 3 h; (**o**) R_1_-CONHNH_2_, DMF, 55 °C, and 3 h; (**p**) aqueous KOH or NaHCO_3_, 100 °C, and 3 h.

**1,2,4-triazole-3-thione (TZT) derivatives:** TZT was identified as a potential motif that interacted with the two zinc ions of MBL (L1) through one of the three nitrogen atoms, and sulfur atom, at the same time. An initial crystal structure evaluation of the analogous compound in complex with the L1 enzyme [132] led to synthesis [133,134,135,136,137,138,139,140] (Scheme 36) and enzymatic inhibition studies on a variety of compounds with variable substituents at positions 4 and 5 of TZT. A number of selected compounds exhibited IC_50_ values in the micro-molar range against representative MBLs (i.e., L1, VIM-4, VIM-2, NDM-1, and IMP-1 and CphA); however, limited success was observed in the microbiological assays [134,135,136,137,139,140]. In addition to the antimicrobial activity against class B β-lactamases, a series of compounds was assessed in vitro against KPC-2 as well. The results indicated the weak but cross-class potential of this motif when selected derivatives were tested in combination with meropenem against clinical strains [138].

**Binding modes of BLIs:** The molecular diversification of the β-lactamases pose challenges for drug researchers when designing broad spectrum BLI. Structural diversification is pronounced in MBLs, which are significantly different from SBLs due to the presence of one or two zinc ions in the active site. The zinc ions are actively responsible for the catalytic hydrolytic activity of the MBLs. All of the MBL enzymes, except the enzymes expressed in *Aeromonas* species that can hydrolyze carbapenems, require two zinc ions for catalytic activity. Crystal structures of representative MBLs such as IMP, VIM, and NDM enzymes confirm that the active site of these MBLs is quite shallow and is close to the surface, in contrast to SBLs, where the active site is deeper and more enclosed [141]. A common catalytic mechanism of di-Zn MBLs proposes that Zn1 and Zn2 simultaneously coordinate to the carbonyl and the exocyclic carboxylate of the incoming substrate antibiotic β-lactam ring. This interaction polarizes the β-lactam carbonyl, and anchors the substrate in the active site of the enzyme. The BL carbonyl is next attacked by the Zn-coordinating OH, forming a predicted tetrahedral adduct. This intermediate collapses to open the ring, generating a nitrogen anion, which is stabilized by Zn2. The protonation of the anion followed by the release of the product and the rebinding of a water molecule completes the catalytic cycle. A notable feature of this mechanism, in contrast with SBLs, is that amino acid residues do not directly participate in the catalytic activity [141,142,143].

There are two major strategies that are adopted to inhibit MBLs. One of them relies on targeting the zinc ions of the enzyme by binding, covalent bonding, or sequestering. The other strategies are independent of targeting the metal ions, and include allosteric inhibition and inhibition by binding the catalytic amino acid residues in the active site [141,144]. Various classes of compounds have been identified as being inhibitors of MBLs, including carboxylic acids, sulphonic acids, and thiol-containing as well as phosphonic acids, each of which possess a distinct inhibition profile and mode of action. In the next section, we will highlight the binding modes of prominent classes of compounds with representative SBLs and MBLs.

**Inhibition modes of cyclic boronates:** The crystal structures of the complexes of a number of β-lactamases in combination with various cyclic boronates were solved by X-ray crystallography. Common features involved in the binding interactions of BLIs and BLAs were identified in the crystal structure in addition to several interactions that are specific to each enzyme–inhibitor complex.

**Figure 3:**
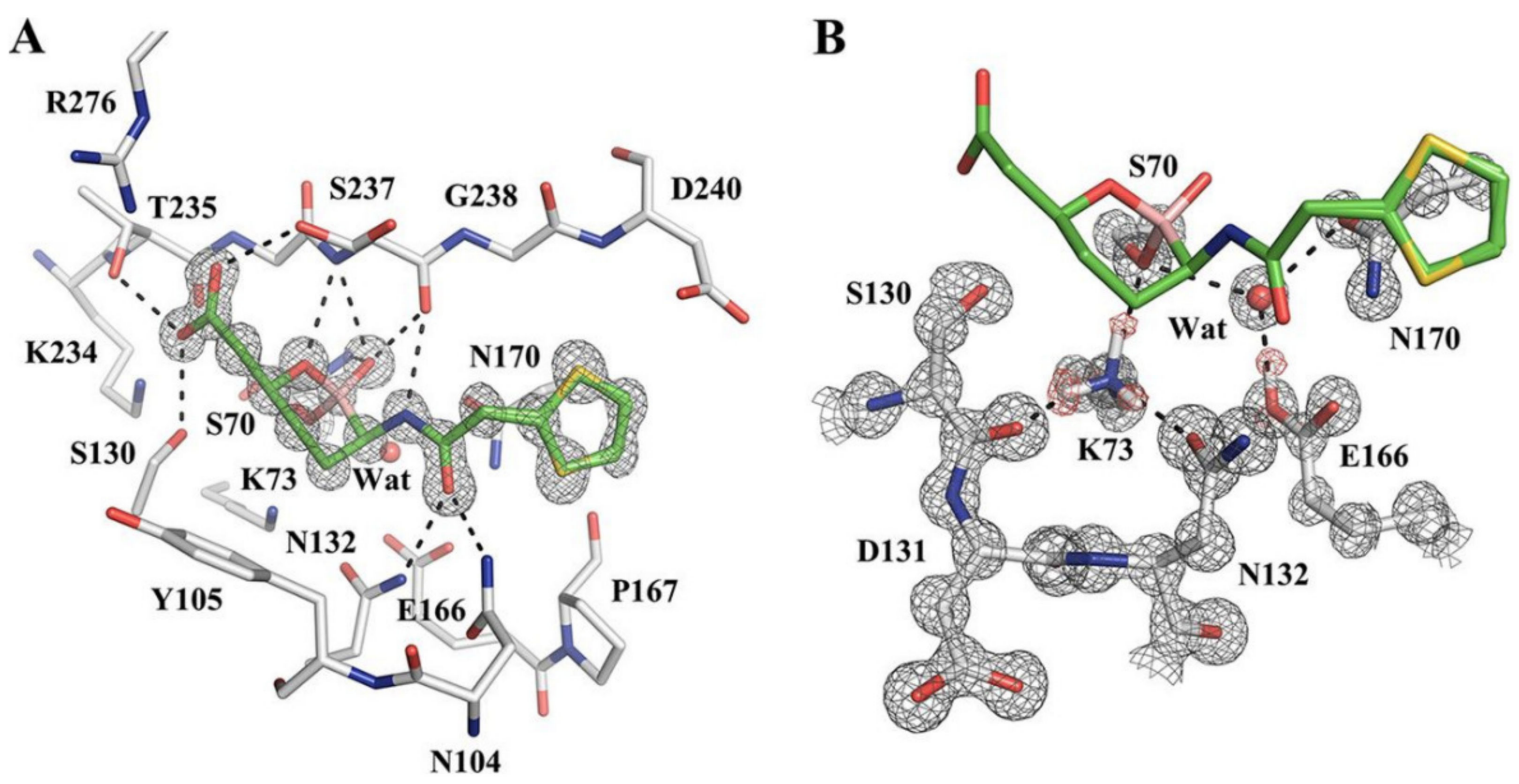
Vaborbactam complex crystal structure with CTX-M-14 class A β-lactamase. (**A**) Vaborbactam covalently bound in the active site of CTX-M-14 (1.0-Å resolution; PDB code 6V7H). The unbiased F_o_-F_c_ omit map of vaborbactam is contoured at 3σ and represented with gray mesh. Hydrogen bonds are shown as black dashed lines. (**B**) Protonation state of K73 and E166 in the CTX-M-14–vaborbactam complex. The 2F_o_-F_c_ omit map (gray) is contoured at 3σ around the protein residues, and the unbiased F_o_-F_c_ omit map (red) is contoured at 2σ around K73 and E166, corresponding to hydrogen atoms located on the terminal groups of K73 and E166. Reprinted with permission from Ref. [145].

**Binding modes of vaborbactam:** Crystal structures of vaborbactam complexed with class A KPC-2, CTX-M-14 (Figure 3) [145], CTX-M-15, and class C AmpC, [32] reveal a reversible covalent bond between the boron atom of the vaborbactam and the serine active site of the enzyme, forming a tetrahedral intermediate. In CTX-M enzymes-inhibitor complexes [32,145], common features include the hydrogen bonding between NH of the amide group of the inhibitor, and S237, N132, and N104 residues of the enzyme. The carboxylate moiety of the inhibitor coordinates with T235, S237, and S130 side chain hydroxyls. Both boron bound oxygen atoms, endocyclic as well as exocyclic, interact with the oxyanion hole. In AmpC-vaborbactam complex hydrogen bonds are formed between N152, Q120 residues, and NH of the amide of inhibitor, and T316 and S318 and carboxyl group, of the inhibitor [32]. Vaborbactam is present as chair conformation in both, CTX-M-15 and AmpC, complexes. The amide substituent occupies the axial position and carboxyl group is in equatorial position in CTX-M-15 complex, whereas this orientation is opposite in AmpC complex. As a result of this conformational change in AmpC-vaborbactam complex, the hydroxyl group of the inhibitor interacts with the oxyanion hole formed by the NH groups of S64 and SD318, whereas endocyclic oxygen forms hydrogen bond with hydroxyl of Y150 [32].

**Figure 4:**
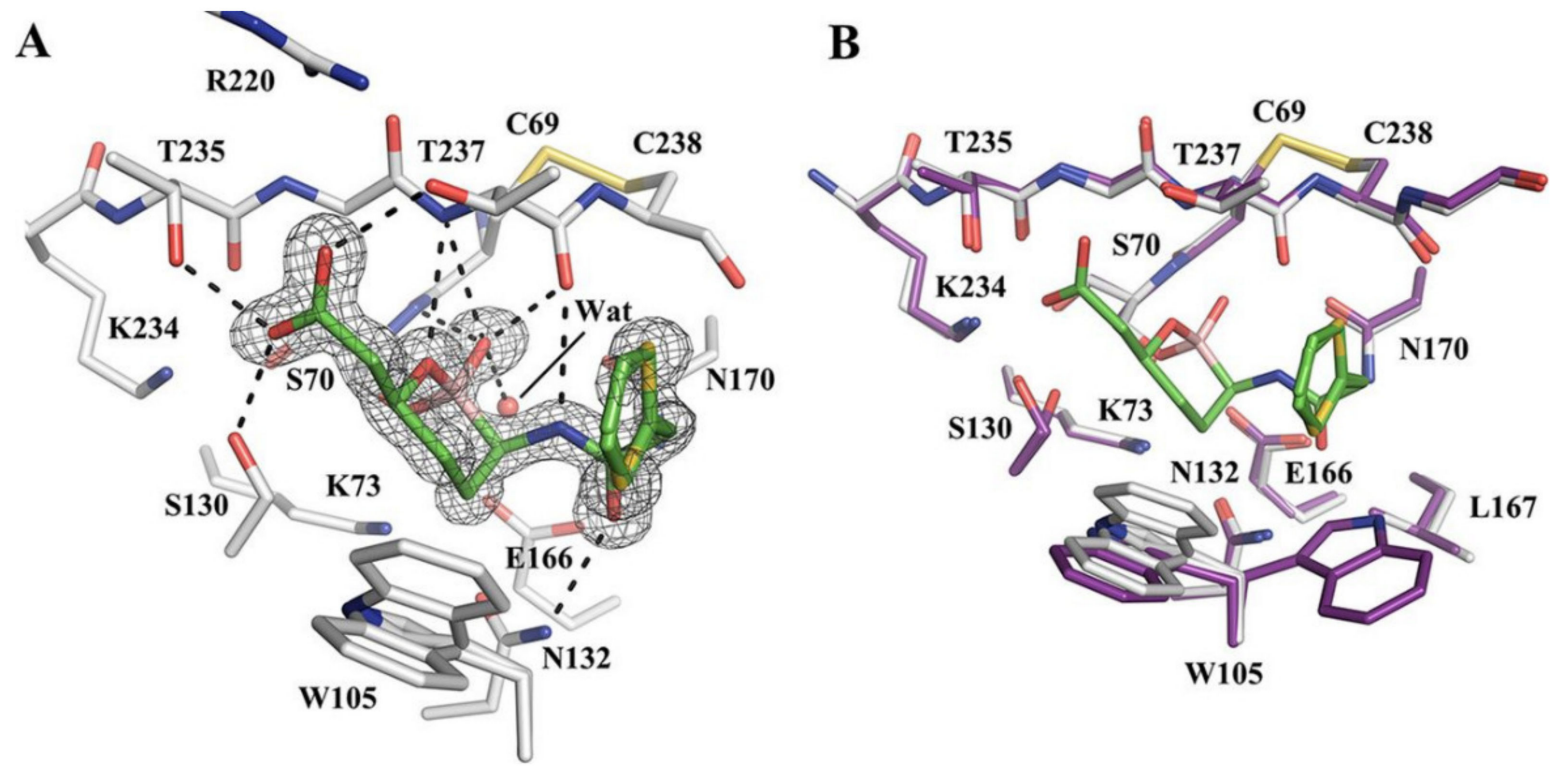
Vaborbactam complex crystal structure with class A carbapenemase KPC-2. (**A**) Vaborbactam covalently bound in the active site of KPC-2 (1.25-Å resolution; PDB code 6V7I). The unbiased F_o_-F_c_ omit map of vaborbactam is contoured at 3σ and represented with gray mesh. Hydrogen bonds are shown as black dashed lines. (**B**) Superimposition of KPC-2–vaborbactam (gray) and KPC-2 apo (PDB code 5UL8; purple) structures Reprinted with permission from Ref. [145].

Hydrogen bonding interactions in KPC-2-vaborbactam (Figure 4) are similar to that of CTX-M-14-vaborbactam complex, except one main difference where KPC-2 T237 residue forms a hydrogen bond with the carboxylate group of inhibitor, in contrast with S237 in CTX-M-14 [145]. Furthermore, it has been established that the inhibition process does not lead to the degradation of the inhibitor in complexation with KPCs, indicating the stability of the inhibitor [100].

**Figure 5:**
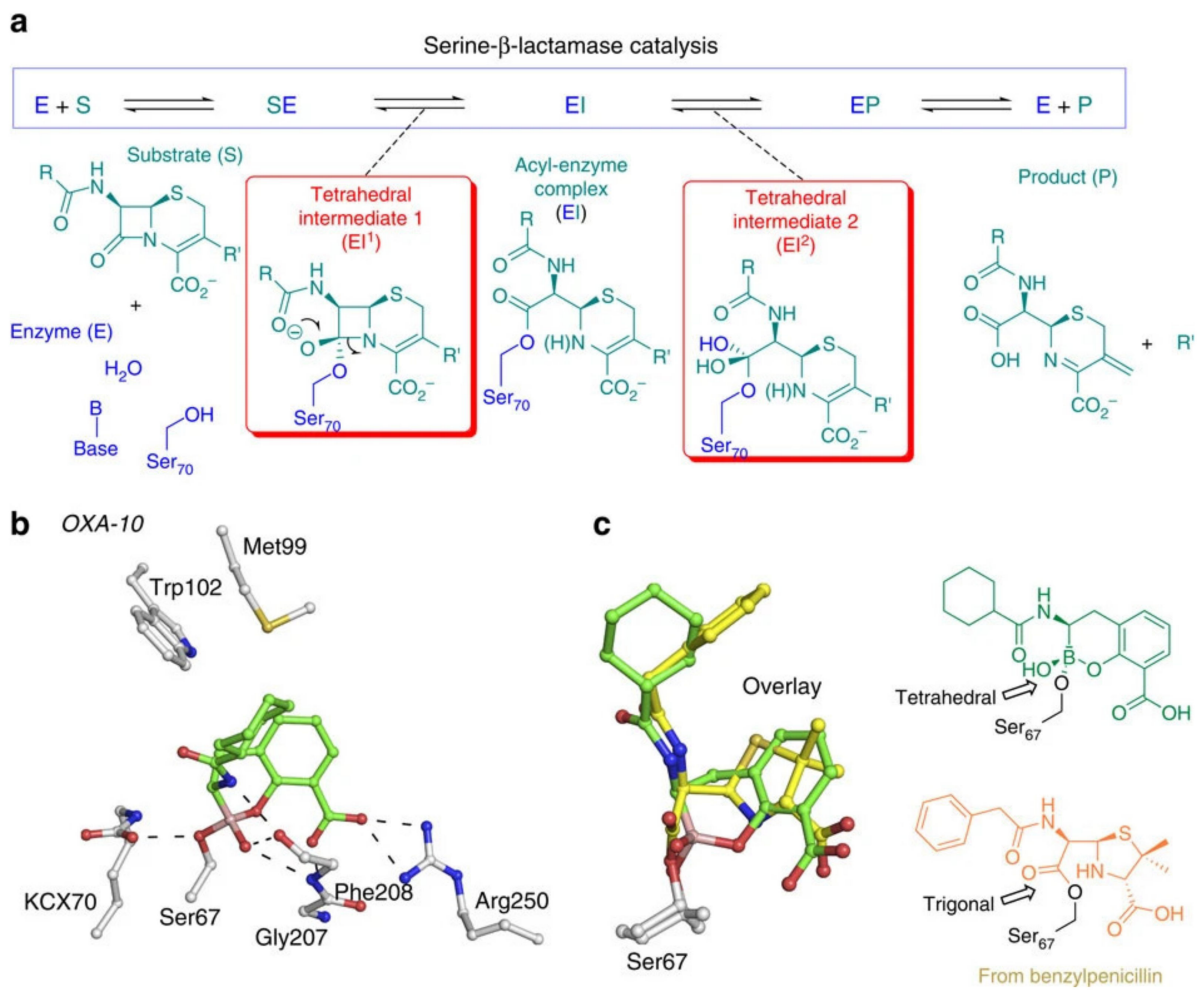
Mode of action of serine-β-lactamases and binding mode of cyclic boronates. (**a**) Outline the mode of action of SBLs. (**b**) View from a structure obtained by the co-crystallization of representative cyclic boronate with OXA-10 (chain A) revealing the binding mode of cyclic boronates to SBLs. (**c**) The overlay compares the binding modes of cyclic boronate and hydrolyzed benzylpenicillin in aa complex with OXA-10 (PDB ID: 2WGI). Reprinted from reference [104].

**Figure 6:**
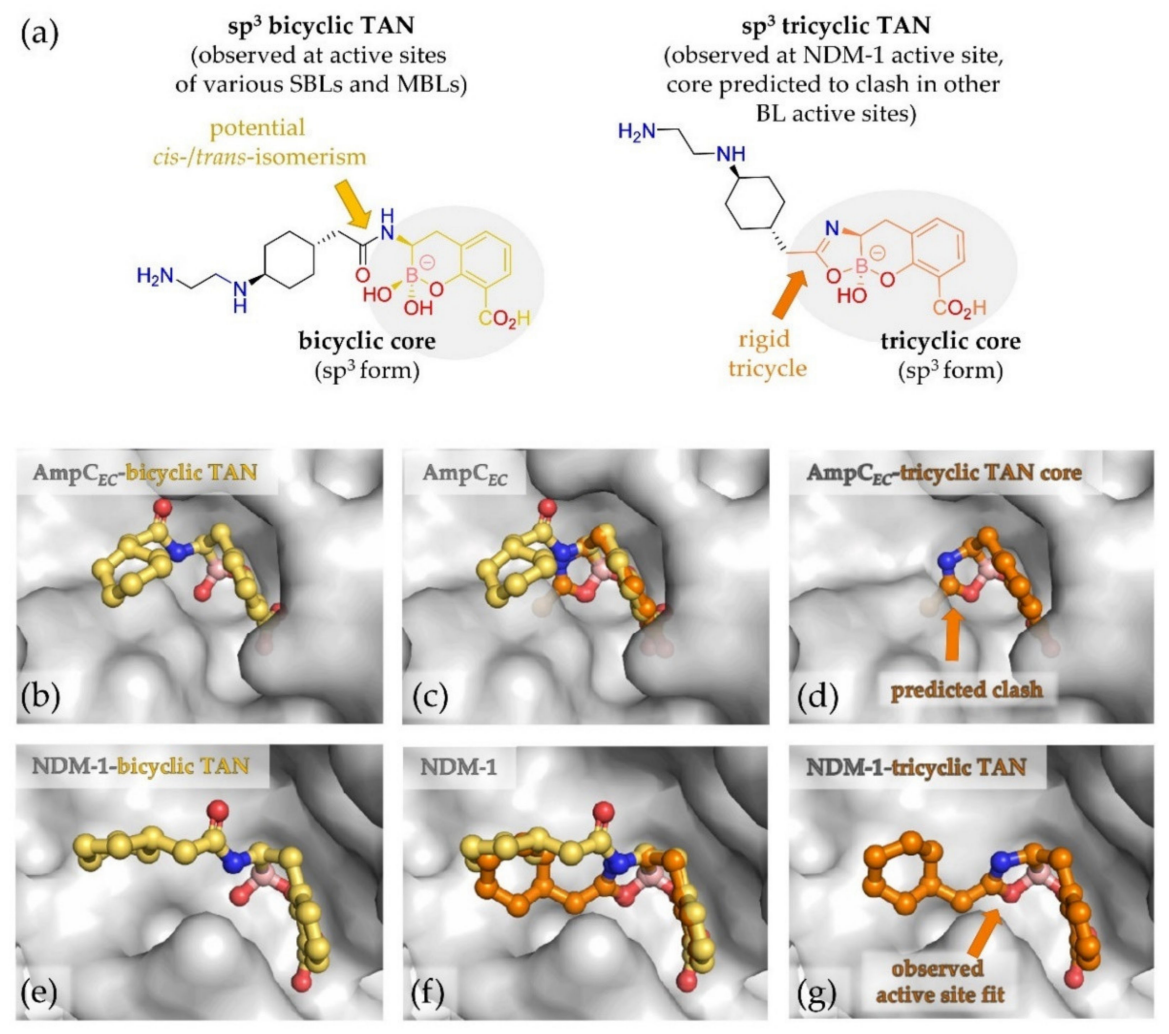
Tricyclic TAN (taniborbactam) likely cannot bind at the AmpC*_EC_* active site due to a steric clash of the putative tricyclic core and side chain. (**a**) Structural comparison of tricyclic and bicyclic forms of TAN; (**b**–**g**) overlay of tricyclic TAN core (orange, not crystallographically observed at the active AmpC*_EC_* site) with bicyclic TAN (yellow) crystallographically observed as being bound to active site S64 of AmpC*_EC_* (PDB ID: 6YEN), revealing a likely steric clash of the rigid tricycle in the AmpC*_EC_* active site as well as the active sites of other β-lactamases. In contrast, both tricyclic (orange) and bicyclic (yellow) forms of TAN have been crystallographically observed at the active site of the B1 MBL NDM-1 (PDB ID: 6RMF). Note, in both structures, the terminal amine of the side chain was disordered and was therefore excluded from the model; (**b**) observed conformation of bicyclic TAN at the AmpC*_EC_* active site; (**c**) alignment of the tricyclic TAN core to bicyclic TAN observed at the AmpC*_EC_* active site; (**d**) putative steric clash of tricyclic TAN in the AmpC*_EC_* active site based on the overlay in (**c**). Note that the apparently flexible parts of the side chain are not shown but would result in a clear steric clash with the active site; (**e**) observed conformation of bicyclic TAN at the active NDM-1 site; (**f**) overlay of tricyclic TAN and bicyclic TAN, both as observed at the active NDM-1 site; (**g**) observed conformation of tricyclic TAN at the active NDM-1 site. Reprinted from reference [146].

**Binding modes of cyclic boronates towards SBLs:** Cyclic boronates such as taniborbactam and **QPX7728** are regarded as pan spectrum BLIs due to their capability of inhibiting both SBLs and MBLs. Kinetics studies using selected SBLs with **QPX7728** revealed a non-covalent interaction between the enzyme and the inhibitor at the onset of enzyme inhibition activity. The subsequent formation of enzyme–inhibitor complex through a covalent bond between the serine residue of the enzyme and the boron atom of the inhibitor suggests a progressive inactivation mechanism [113]. The crystal structures of taniborbactam, **QPX7728**, and analogous cyclic boronates with different SBLs, such as CTX-M-14, KPC-2, OXA-10, (Figure 5) [104] and OXA-48 [114], have been studied for X-ray crystallographic analysis [104,106,113,114]. The crystal structures of these SBLs [114] with **QPX7728** have demonstrated that the serine residue is covalently linked to the boron atom of the inhibitor, which is in agreement with the above-mentioned kinetic model [113]. Taniborbactam crystals bound to various serine β-lactamases, such as OXA-10 [110], OXA-48 [114], AmpC [146], and CTX-M-15 [104,106,146], demonstrate binding interactions similar to **QPX7728**. The covalent linkage of the boron atom of the inhibitor with the serine residue of the enzyme [114] forming a tetrahedral structure mimics the tetrahedral acyl–enzyme intermediate [146]. Further enzyme–inhibitor complex strengthening is affected by the hydrogen bonding interactions of the amide moiety of the inhibitor with the enzyme residues Ser237, Asn132, and Asn104 and between the carboxylate moiety of the inhibitor and the residues Thr235 and Ser237. In case of taniborbactam, side chains and the cyclohexyl moiety occupies the same space where the aryl group of several penicillins and cephalosporins resides, whereas the ethyl diamine appendage is directed toward crystallographic water molecule and has no apparent direct interaction with the enzyme [106]. In another study, taniborbactam was found on the surface of the AmpC enzyme as tricyclic boronate (Figure 6) in addition to a bicyclic structure that was present in the active site of the enzyme [146].

**Binding modes of cyclic boronates towards MBLs:** Kinetic studies of **QPX2278** with various MBLs such as NDM-1, VIM-1, and IMP-1, reveal linear inactivation compared to progressive inactivation in combination with SBLs. The fast equilibrium formation between the inhibitor and enzyme is typical for the reversible “fast on–fast off” mechanism. Fast and reversible equilibrium is consistent with the non-covalent complexation of **QPX7728** with NDM-1 and VIM-1 MBLs [113,114]. The crystal structures of cyclic boronate complexes with MBLs have been extensively reported. The structures of NDM-1, BcII, and VIM-2 in complex with cyclic boronates indicate that both boron-bound oxygen atoms participate in the bidentate coordination of the Zn1 ion of the enzyme. Oxygen atoms of the inhibitor carboxylate coordinate to Zn2; the other carboxylate oxygen interacts with Lys224 (NDM-1 and BcII) or Arg228 (VIM-2) by means of hydrogen bonding/electrostatic interactions. The endocyclic boronate ester oxygen coordinates to Zn2 [104]. Slight variations in these interactions were observed in the case of NDM-1–taniborbactam complex, instead of its original bicycle form, the inhibitor was observed as tricyclic structure (Figure 6) formed by the cyclization of the amide oxygen onto the boron atom [110,146]. This observation suggests that boronate-based BLIs are able to adapt multiple forms when interacting with different enzymes. In addition, crystal structure of this complex also revealed that one boron-bound oxygen effectively bridges the two active site zinc ions and is closer to Zn1 than Zn2, therefore resulting in a significantly weaker interaction with Zn2. Other taniborbactam interactions are conserved with respect to other bicyclic boronates, such as the interaction of aryl carboxylate with Zn2 and Lys224 and the “endocyclic” boronate ester oxygen with Zn2 [110]. Overall orientation of taniborbactam in NDM-1 and VIM-2 was identical to that of SBLs with slight difference that carboxylate is nearly co-planar in MBLs whereas orthogonal in SBLs. Polar interactions vary across various structures; however, the hydrophobic moieties of the inhibitor are packed against the lipophilic side chains of the enzyme in all cases [114].

**Figure 7:**
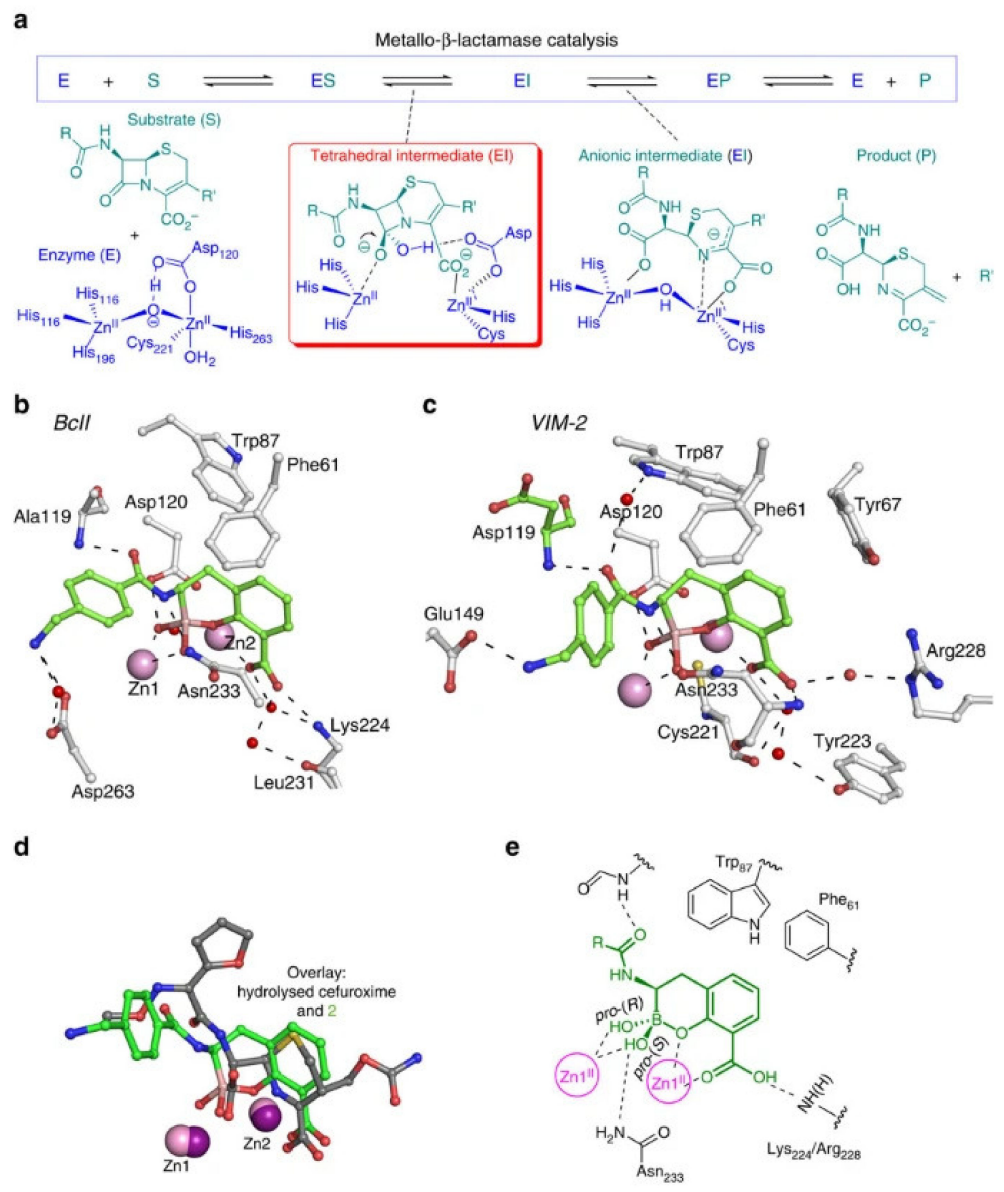
Mode of action of metallo-β-lactamases and binding mode of cyclic boronates. (**a**) Outline mode of action of MBLs showing proposed intermediates. (**b**,**c**) Views from structures obtained by the co-crystallization of representative cyclic boronate with BcII (**b**) and VIM-2 (chain A) (**c**). Note that the relative position of Trp87 is rotated by ∼180° compared to its position in the structures of BcII and VIM-2 without an inhibitor; in the case of VIM-2, Trp87 interacts with the cyclic boronate via a water molecule. (**d**) The overlay compares the binding modes of cyclic boronate and hydrolysed cefuroxime in a complex with NDM-1 (PDB ID: 4RL2)). (**e**) Key active site interactions by the cyclic boronates [104].

In most of the MBL and cyclic boronate complexes, the binding interactions between the oxygen of the inhibitor and the zinc ions of the enzyme lead to an increase in the Zn1−Zn2 distance, and the geometry of the resulting structure resembles the tetrahedral oxyanion formed during β-lactam hydrolysis. This suggests that these inhibitors act as mimics of the MBL/BL–fragment complexes formed by following the nucleophilic attack of the BL carbonyl by the hydroxide anion of the enzyme (Figure 7) [104,146].

Comparing the interactions of the complexes of cyclic boronate with those of SBLs and MBLs, it has been concluded that despite a variety of differing side chain orientations, the binding mode of the fused bicyclic boronate motif was strikingly conserved in both SBLs and MBLs [110]. Additionally, it has been observed that the scaffold with a cyclic boronate fused to a benzoic acid is the key binding motif for pan-β-lactamase inhibition [106].

**Binding interactions of acyclic boronates:** To assess the dual inhibition mode of action of these compounds, crystal structures of the selected derivatives in complex with VIM-2 (MBL) and KPC-2 (SBL) were characterized by X-ray crystallography. It was observed that MBL inhibition involves the thiol-driven metal chelating mode of inhibition, similar to the inhibition mode that was previously observed for other thiol-based inhibitors. In this case, thiol binds to the two active site Zn ions by displacing the zinc-bridging hydroxide. The amido carbonyl interacts with Asn233 on the L10 loop of the VIM-2 through hydrogen bonding, while boronic acid forms a hydrogen bond with Arg228 residue [115].

The KPC-2-inhibitor structure reveals that the inhibitor binds with KPC-2 via the formation of a covalent adduct between its boronic acid and Ser70. This inhibition mode is similar to that of previously reported boronic acid-based SBL inhibitors. In addition, this binding is further stabilized by multiple hydrogen-bonding interactions with catalytically important residues such as Ser70, Thr237, Asn170, Glu166, and Asn132 [115].

**Inhibition modes of BTZs:** The co-crystal structures of the MBLs, including VIM-2 [118], NDM-1 [119], IMP-1, BcII, and L1 [120], with different BTZs have been studied to determine the binding interactions of the inhibitor with the enzyme. Similar to other thiol containing inhibitors, the thiol groups of BTZs also form a bridging interaction with the two zinc ions of the inhibitor by displacing the hydroxide/water molecule, and as such, an increase in the distance between the two zinc ions is observed. Interestingly, carboxylate does not bind to the Zn2; instead, it forms hydrogen bonds with water molecules, forming a bridge between the Zn2 and K224 (IMP-1, NDM-1), Cys221 (VIM-2), and S223 (L1) residues. In contrast to the aforementioned dizinc MBLs, the BTZ thiol is not involved in the interactions with the zinc ion of the mono zinc B2 MBL Sfh-I complexed with BTZ. Instead, the carboxylate group of the BTZ interacts with the active-site zinc, displacing the water, maintaining the tetrahedral zinc geometry, and causing the H196 side chain to flip. This movement results in a loss in the interaction of H196 with the nucleophilic water, which leads to the inactivation of the enzyme [118,119,120].

**Binding interactions of MMTZs:** MMTZ binding involves the interaction of the thiol directly with the monozinc center of Sfh-I in contrast to BTZs or bridging the dizinc center of L1 and displacing the catalytic water/hydroxide. In Sfh-I, there is a weak hydrogen bond between the ethyl ester carbonyl of MMTZ and the side chain nitrogen of Asn233. In contrast, L1 binding is stabilized by stronger hydrogen bonds with the oxygen side chains of Ser223 (2.5 Å) and Tyr33 (2.9 Å). [121]. In addition to the thiol coordination of the zinc ions at the active site, extensive hydrophobic interactions between enzyme residues and the inhibitor lead to the deeper localization of the inhibitor within the active site. Unexpectedly, MMTZ binding features a thioether–π interaction with a conserved aromatic residue acting as an active site, which is consistent with their equipotent inhibition and similar binding to multiple MBLs [122].

**Interactions of SHCs with MBLs:** In the crystal structures of IMP-1, NDM-1, VIM-1, or VIM-2 complexes with SHCs, the nitrogen atom of the sulfamoyl group coordinates with the Zn1 and Zn2, replacing the hydrolytic water, which is located nearly equidistantly to both metal ions. Asp120 forms hydrogen bonds with the sulfamoyl group, and the carbonyl oxygen of the sulfamoyl group is also hydrogen-bonded to the nitrogen atom of the Asn233 side chain. The spatial position of the Asn233 side chain shifted toward the zinc ions. One carboxylate oxygen coordinates with Zn2, while the other one interacts with the amide of Asn233 and the amino group of either Lys224 (IMP-1, NDM-1) or Arg228 (VIM-2) [123,147].

**Binding modes of ANT431:** ANT431 binding in the enzyme active site relies on the interactions between the thiazole moiety, Zn2, and the Arg228 side chain. The X-ray structure of the VIM-2 bound to ANT431 showed that the sulfonamide substituent is located between residues Phe61 and Tyr67, inducing a significant rotation of the side chain of the later [125].

**Inhibition modes of InCs:** The crystal structures of InCs with VIM-1,VIM-2, NDM-7, and L1 revealed a highly conserved and unprecedented MBL binding mode for the InCs. The C2 of the InC carboxylate ligates to Zn2, enabling InCs to engage with the different motifs employed in MBL–substrate carboxylate binding; for example, VIM-1 uses a histidine-, VIM-2 uses an arginine-, NDM-7 uses a lysine, and L1 uses a serine residue [131].

**Figure 8:**
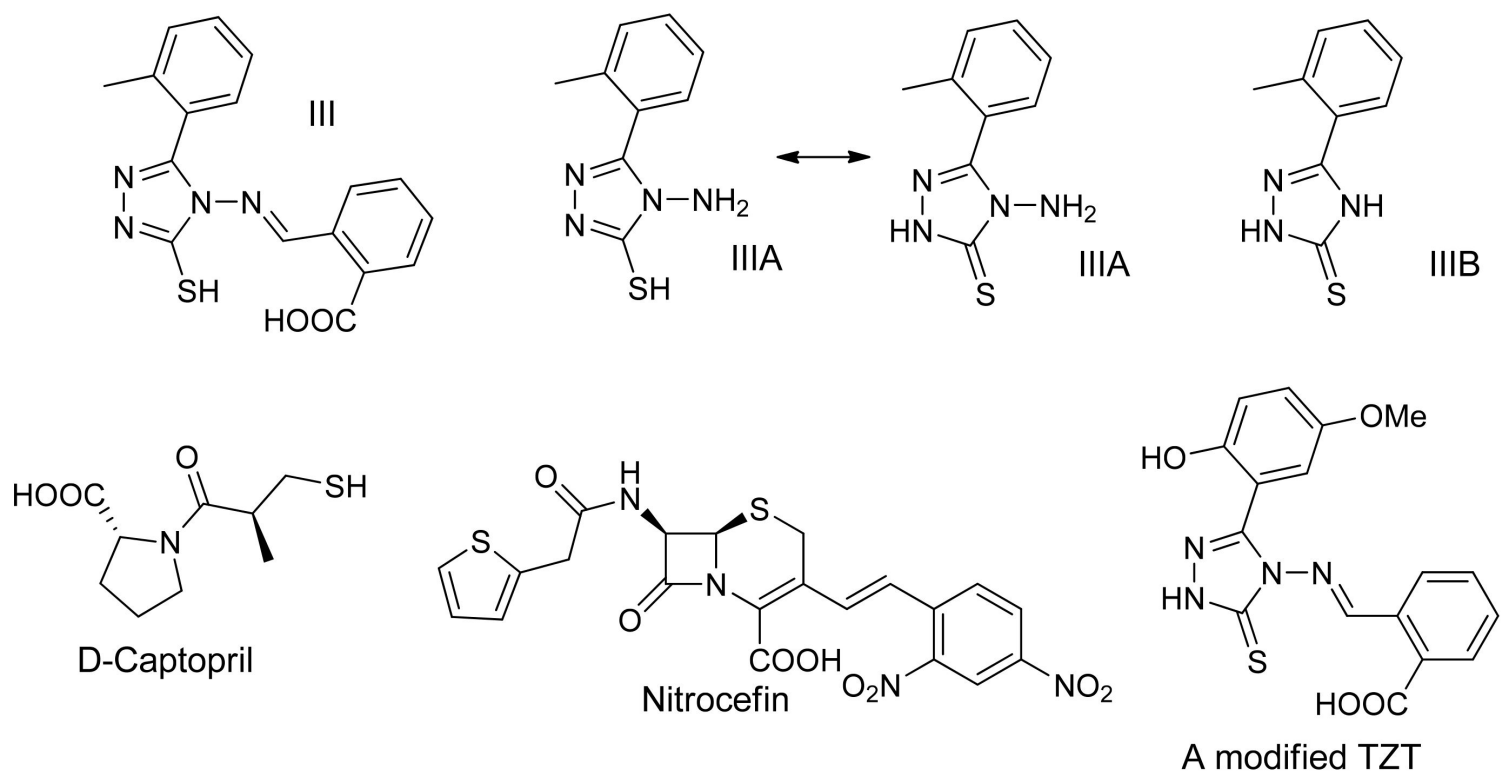
Structures of D-captopril, nitrocefin, and TZTs used for binding with L1 (MBL).

**Figure 9:**
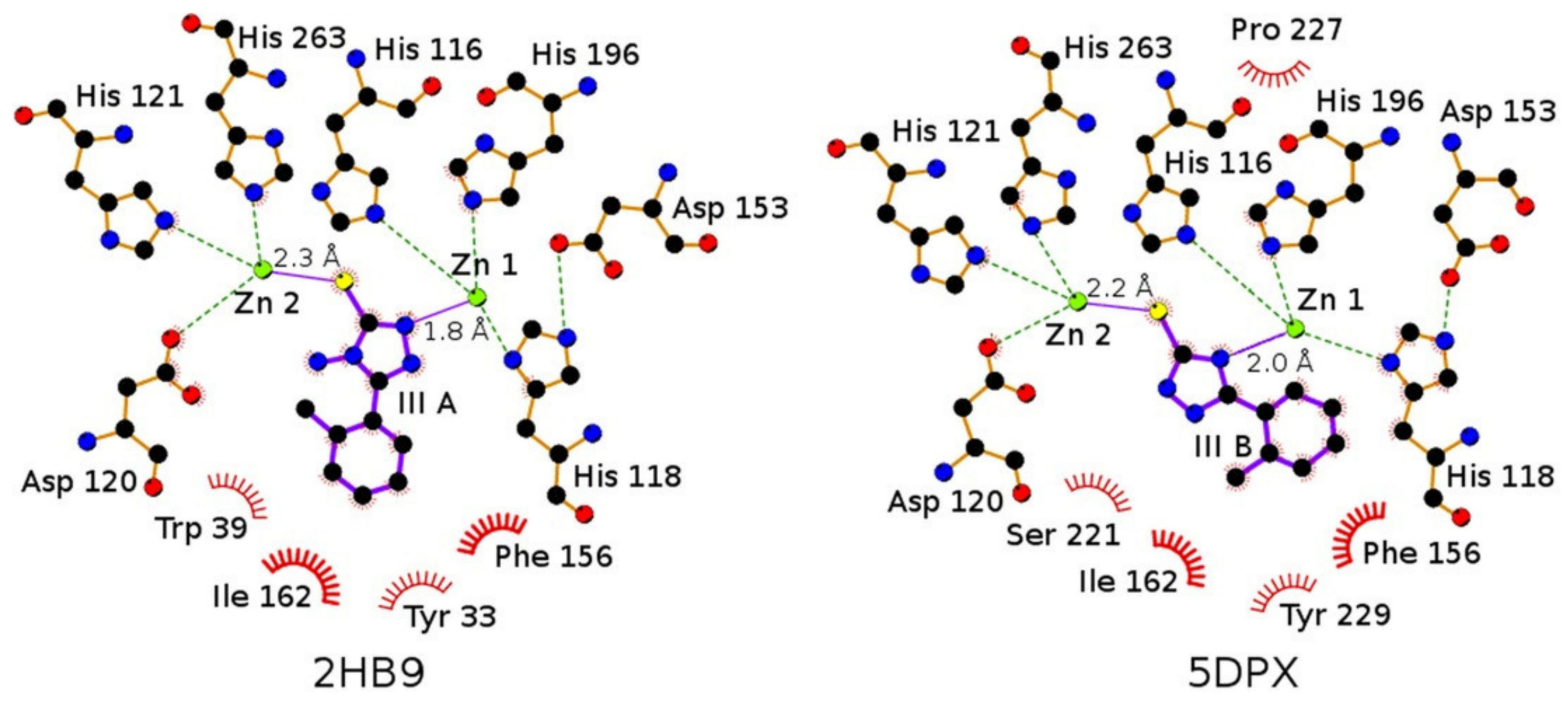
Binding mode of the 1,2,4-triazole-3-thione moiety of compounds IIIA and IIIB in the dizinc active site of L1. Reprinted with permission from Ref. [134] Wiley-VCH.

**Figure 10:**
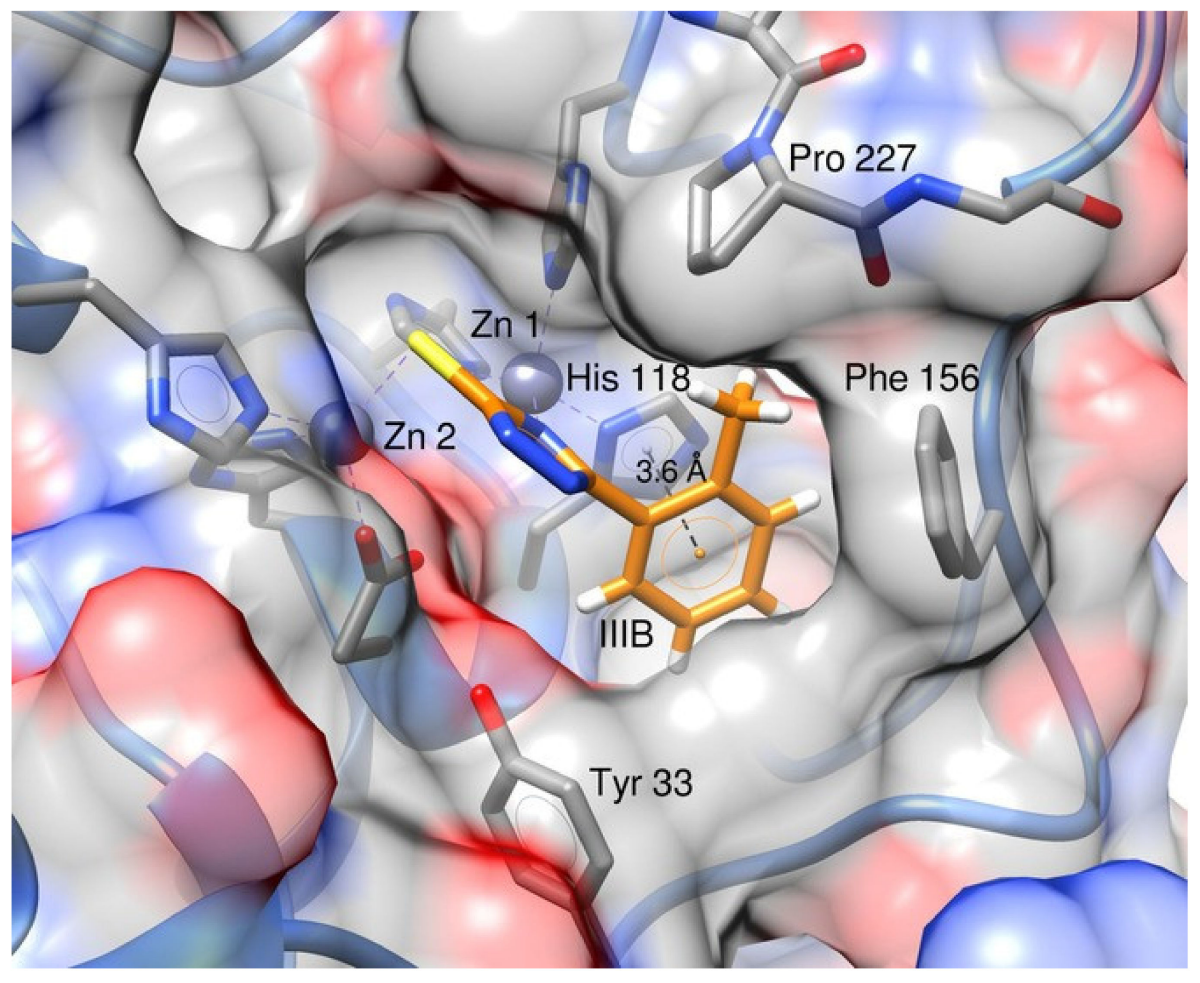
Compound IIIB in the active site of L1 from 5DPX.pdb. The image shows the interactions established with the two Zn ions and π stacking with His118. Reprinted with permission from Ref. [134] Wiley-VCH.

**Inhibition modes of TZT:** The TZT scaffold was developed from the findings of crystallographic studies carried out by Nauton [148] and Olsen [132] et al. on the complexes of L1 with nitrocefin, D-captopril, MP2, and three other structures (Figure 8) with carboxyl, thiol, and thiazole functionalities. The crystal structure data revealed that the compounds bind with the zinc ions through bridging by replacing the hydroxide of the water molecule, except for compound IIIA, the derivative of compound III [132]. According to these studies, it was affirmed that the binding of the IIIA with L1 led to the distortion of the zinc center, with the distance increasing from 3.5 to 4.6, and Zn1 forms a tetrahedral geometry with the nitrogen of the triazole ring, while sulfur binds with Zn2. The NH2 group interacts with Asp120, and the phenyl ring has a weak CH–π interaction with a Ile162 methyl group [148]. The simultaneous interaction of an inhibitor with two zinc ions led to the exploration of analogous compounds. Following the synthesis and crystallographic analysis of compound IIIB, an analog of IIIA that is devoid of an extra-triazole amino group in IIIA, a complex with L1 showed slightly different interactions with L1. IIIB was also able to bind with two zinc ions but maintained a slightly different pattern than IIIA; in this case, N4 (instead of N2) interacted with Zn1 and S3 with Zn2, and an aromatic phenyl ring formed a strongly stabilizing π stacking interaction with His118. The compound IIIB also showed increased inhibition activity compared to IIIA, indicating that the NH_2_ group is not necessary for inhibition (Figure 9 and Figure 10) [134].

Furthermore, the crystal structure of the analogs of IIIA complexed with VIM-2 showed N2 coordinating with Zn1 and that Zn2 was interacting with the inhibitor S3, displacing the catalytic hydroxide ion. In addition, several active site residues, the methoxy and hydroxy oxygen atoms of the substituent at position 5 in particular, were H-bonded to the NH of Trp87 and NH_2_ of the conserved Asn233, carboxylate was H-bonded to the backbone NH of Asn233 and two water molecules, and the aromatic rings also interacted with the side chains of Phe61 and Tyr67 with the establishment of T-shaped π stacking interactions [137].

## 2. Conclusions and Future Perspective

Four classes of β-lactams namely, penicillins, cephalosporins, carbapenems, and monobactams, shared the highest percentage (65%) of antibiotic prescriptions in United States during the period from 2004 to 2014 [149]. The success of this class of antibiotics is the frequent introduction of new drugs from time to time for the antibacterial treatment of almost all kinds of bacterial infections. However, with the emergence and exponential increase in β-lactamases, especially carbapenemases, the need for new antibiotics has been enhanced. The WHO recently categorized the bacterial pathogens for remedial R&D, indicating that *Acinetobacter baumannii*, *Pseudomonas aeruginosa,* and *Enterobacteriaceae* are the highest-priority pathogens [150]. The urge for the development of focused remedies against these bacteria requires the discovery of new antibiotics and the development of new BLIs that are capable of inhibiting SBLs as well as MBLs altogether.

As far as DBOs are concerned, after the successful clinical introduction of avibactam and relebactam, several other derivatives of avibactam and relebactam are under clinical investigations in efforts to increase the spectrum of inhibition of this class of compounds [151,152]. In addition to DBOs, boronates are important motifs for the β-lactamase inhibition program which has furnished vaborbactam as second-generation non-β-lactam-based BLI, and more cyclic boronates such as taniborbactam and QPX7728 are now in phase III and phase I clinical trials, respectively, against B1 MBLs [151,152,153]. The recently approved BLIs are effective against a large array of SBLs; however, they are not effective against MBLs. Therefore, it is still necessary that the research focus should be diverted to the development of MBLs. In this regard, besides DBOs and cyclic boronates, several other motifs have been identified, and have been targeted towards the binding efficiency of those motifs with zinc ions of MBLs. These efforts have led to the introduction of ANT-2681, a thiazole carboxylate derivative, into the clinical trials in combination with diverse number of antibiotics [151]. In addition, a few classes of compounds have demonstrated cross-class inhibition ability against selected SBLs and MBLs. This provides directions towards a promising area of research for the future design of wide-spectrum-inhibitor scaffolds.

The emergence of new pathogenic strains with β-lactamases that are resistant against BLs and recently reported [66,154,155] BL–BLI combinations, e.g., ceftazidime–avibactam, indicates that continuous efforts to find new combinations should be maintained at an increased pace. Alternatively, new avenues of β-lactamase inhibition should be explored, spanning from diverse classes of compounds, that are capable of restoring the efficacy of susceptible antibiotics against all classes of BLAs.

## Figures and Tables

**Table 1:** Antimicrobial agents based on the mechanism of action and modes of resistance [2,8].

Mechanism of Action	Antimicrobial Groups (Antibiotic/s)	Mode(s) of Resistance
Inhibit Cell Wall Synthesis	β-Lactams	Hydrolysis, efflux, altered target
	Carbapenems (meropenem) Cephalosporins (cephamycin) Monobactams (aztreonam) Penicillins (ampicillin)	
	Glycopeptides (vancomycin, teicoplanin)	Reprogramming peptidoglycan biosynthesis
Depolarize Cell Membrane	Lipopeptides (daptomycin)	Altered target
Inhibit Protein Synthesis	Bind to 30S Ribosomal Subunit	Phosphorylation, acetylation, nucleotidylation, efflux, altered target
	Aminoglycosides (gentamicin, streptomycin)	
	Tetracyclines (minocycline, tigecycline)	Monooxygenation, efflux, altered target
	Bind to 50S Ribosomal Subunit	
	Phenicols (Chloramphenicol)	Acetylation, efflux, altered target
	Lincosamides (clindamycin)	Nucleotidylation, efflux, altered target
	Macrolides (erythromycin, azithromicin)	Hydrolysis, glycosylation, phosphorylation, efflux, altered target
	Oxazolidinones (linezolid)	Efflux, altered target
	Streptogramins (synercid)	C-O lyase (type B streptogramins), acetylation (type A streptogramins), efflux, altered target
Inhibit Nucleic Acid Synthesis	Quinolones (ciprofloxacin)	Acetylation, efflux, altered target
	Fluoroquinolones	
Inhibit Metabolic Pathways	Sulfonamides (sulfamethoxazole)	Efflux, altered target
	Pyrimidines (Trimethoprim)	Efflux, altered target

**Table 2:** Classification of β-lactamases, important pathogens, and inhibitors currently in use against β-lactamases [28,29,30].

Ambler	Bush	Substrate β-Lactam	Common Enzymes	Common Pathogens	Effective BLI
A	2a	Penicillins	PC1	*S. aureus*	Clavulanic acid, sulbactam, tazobactam
A	2b	Penicillins and early cephalosporins	TEM-1, TEM-2, SHV-1	*E. coli, K. pneumoniae*	Clavulanic acid, sulbactam, tazobactam, avibactam
A	2be	Extended-spectrum cephalosporins, monobactams	TEM-2, SHV-2, CTX-M-15, PER-1, VEB-1,	*E. coli, Klebsiella spp., Proteus spp.*	Clavulanic acid
A	2br	Penicillins	TEM-30, SHV-10	*E. coli, Klebsiella spp., Proteus spp.*	Avibactam
A	2ber	Extended-spectrum cephalosporins, monobactams	TEM-50		Avibactam
A	2c	Carbenicillin	PSE-1, CARB-3		Clavulanic acid
A	2ce	Carbenicillin, cefepime	RTG-4		Clavulanic acid
A	2e	Extended-spectrum cephalosporins	CepA	*E. coli, B. fragilis, E. cloacae*	Clavulanic acid
A	2f	Carbapenems	KPC-2, IMI-1, SME-1	*K. pneumoniae, Serratia spp.*	
B	3a	Carbapenems	IMP-1, VIM-1, IND-1, CerA, NDM-1	*P. aeruginosa, K. pneumoniae, A. baumannii, S. maltophilia*	
B	3b	Carbapenems	CphA, Sfh-1		
C	1	Cephalosporins	AmpC, P99, ACT-1, CMY-2, FOX-1, MIR-1	*Enterobacter spp., E. coli, P. aeruginosa, Serratia spp., C. freundii*	
C	1e	Cephalosporins	GC-1, CMY-37		Tazobactam
D	2d	Cloxacillin	OXA-1, OXA-10	*K. pneumoniae, E. coli, A. baumannii*	
D	2de	Extended-spectrum cephalosporins	OXA-11, OXA-15		Avibactam
D	2df	Carbapenems	OXA-23, OXA-48		Avibactam

**Table 3:** Classification of carbapenemases and ESBLs.

Carbapenemases [31,32]					Extended Spectrum β-Lactamases [27,33,34]
Serine Carbapenemases		Metallo-Carbapenemases			Serine β-Lactamases
Class A	Class D	B1	B2	B3	Class A
KPC, SME, NMC-A, IML, GES-2	OXA-23, OXA-24/40, OXA-48	IMP, VIM, NDM	CphA	L1C	TEM, SHV, CTX-M, VEB, PER, IRT, CMT, GES, BEL, TLA, SFO
					Class D
					OXA-11, OXA-14, OXA-15, OXA-17

## Data Availability

Not applicable.
